# Consistent condom use among highly effective contraceptive users in an HIV-endemic area in rural Kenya

**DOI:** 10.1371/journal.pone.0216208

**Published:** 2019-05-06

**Authors:** Hodaka Kosugi, Akira Shibanuma, Junko Kiriya, Sam W. Wafula, Masamine Jimba

**Affiliations:** 1 Department of Community and Global Health, Graduate School of Medicine, The University of Tokyo, Tokyo, Japan; 2 School of Public Health, University of Nairobi, Nairobi, Kenya; University of California San Diego, UNITED STATES

## Abstract

**Background:**

Women of reproductive age are at the highest risk of both HIV infection and unintended pregnancy in sub-Saharan Africa. Highly effective contraceptives (HECs) such as hormonal injectable and implants are widely used in this region. HECs are effective for preventing pregnancies. However, unlike condoms, HECs offer no protection against HIV. Dual-method use, or the use of condoms with HECs, is an ideal option to reduce HIV risk but is infrequently practiced. Rather, women tend not to use condoms when they use HECs and increase their HIV risk from their sexual partners. However, it remains unknown whether HIV status affects such tendency. Given the increasing popularity of HECs in sub-Saharan Africa, this study examined the association between the use of HECs and condom use among HIV-positive and negative women.

**Methods:**

A cross-sectional study was conducted among 833 sexually active women aged 18–49 years, recruited from six clinics in Siaya county, Kenya. From March to May 2017, female research assistants interviewed the women using a structured questionnaire. Multiple logistic regression analysis was conducted to examine the association between HEC use and consistent condom use in the past 90 days, adjusting for potential confounders. It was also examined with regular partners (husbands or live-in partners) and non-regular partners, separately. In addition, a sub-sample analysis of HIV-negative or unknown women was conducted.

**Results:**

In total, 735 women were available for the analysis. Among the women, 231 (31.4%) were HIV-positive. HIV-positive women were more likely to use HECs than HIV-negative or status unknown women (70.1% vs. 61.7%, p = 0.027). HEC use was significantly associated with decreased condom use with a regular partner (adjusted odds ratio (AOR) = 0.25; 95% CI 0.15–0.43, p<0.001) and a non-regular partner (AOR = 0.25; 95% CI 0.11–0.58, p = 0.001). However, compared with HIV-negative or status unknown women, HIV-positive women were more likely to use HECs and condoms consistently with a regular partner (AOR = 6.54, 95% CI 2.15–20.00, p = 0.001). Other factors significantly associated with consistent condom use included partner’s positive attitude toward contraception, partner’s HIV-positive status, high HIV risk perception, and desire for children in the future.

**Conclusion:**

Dual-method use was limited among HIV-negative women and women who had HIV-negative partners due to inconsistent condom use. The use of HECs was significantly associated with decreased condom use, regardless of partner type and their HIV status. Due to this inverse association, HIV-negative women may increase their HIV risk from their sexual partners. Therefore, interventions should be strengthened to reduce their dual risks of HIV infection and unintended pregnancy by promoting dual-method use. Family planning services should strengthen counseling on the possible risk of HIV infection from their sexual partners and target not only women but also their partners, who may play a key role in condom use.

## Background

HIV infection remains a global health issue, and 36.7 million people are living with HIV worldwide in 2016 [1]. Sub-Saharan Africa bears the greatest burden of the HIV epidemic. Among the global population with HIV, 72.2% resided in this region, and an estimated 1.2 million people were infected with HIV in 2016 [1]. In sub-Saharan Africa, women are disproportionally affected by HIV, and they accounted for 58.3% of people living with HIV in 2016 [1]. This gender disparity starts when women reach their reproductive age [2]. The main route of HIV transmission is via heterosexual sex in this region [2, 3], and women are infected with HIV at least five to seven years earlier compared to men [2].

Highly effective contraceptives (HECs), such as hormonal injectable and implants, have been reported to increase the risk of HIV infection [4]. In a cohort study of HIV-serodiscordant couples in seven sub-Saharan African countries, the rates of HIV acquisition were almost twice as high among women who used hormonal injectable as those who did not [4]. HEC users can increase the risk of HIV infection due to changes in the immune system, genital tract flora, and HIV receptors [5, 6]. Furthermore, by utilizing HECs, women may be inclined to decrease the use of barrier methods such as male and female condoms. HECs are the most reliable methods for preventing pregnancy. However, unlike condoms, HECs do not prevent HIV infection [7, 8]. In the United States (USA), condom use was lower among women who used HECs than those who did not [9, 10]. This trade-off may increase the risk of HIV infection among women of reproductive age, especially in high HIV prevalence settings.

Dual protection is defined as a protection against the dual risks of unintended pregnancy and STIs including HIV [11]. Protection can be accomplished by either using condoms consistently or together with HECs (dual-method use) [11]. However, dual-method use has been recommended more as condom use alone can only prevent 85% of unintended pregnancy due to incorrect and inconsistent use [11]. Nevertheless, it has been insufficiently practiced in sub-Saharan Africa [11,12]. Among HIV-positive women, only 15.7% and 27.2% utilized this method in Ethiopia and Nigeria, respectively [11,12]. Moreover, the effect of HIV status on the trade-off between HEC use and consistent condom use remains unknown.

Kenya is one of the countries most affected by the HIV epidemic, with an estimated adult prevalence of 5.9% in 2015 [13]. Like other sub-Saharan African countries, it was higher among women (6.5%) compared to men (4.7%) [13]. Kenya also has marked a substantial increase in the use of HECs during the past decades [14]. Of married or in-union women of reproductive age, 58.0% used HECs in 2015 [15]. HECs, which are the main source of contraceptives, are provided for free in public health facilities [16, 17]. Kenya has promoted condom use as a fundamental strategy to reduce HIV risk [18], which led to a high HIV awareness and knowledge of condoms. According to the Demographic Health Survey 2014, over 99% of women had heard of HIV/AIDS, and almost 80% of women were aware that condoms could prevent HIV infection [17]. Despite the efforts, condom use remains low in Kenya [15]. For example, only 40.0% of women with multiple partners reported using a condom at the last sexual intercourse [17].

Given the increasing popularity of HECs in Kenya, where the HIV epidemic is disproportionally affecting women, this study aimed to examine the associations between the use of HECs and condom use among HIV-positive and negative women.

## Methods

### Study design and settings

A health facility-based cross-sectional study was conducted in Siaya county in Kenya. The study area had the second highest HIV prevalence (24.8%) after Homa Bay county (26.0%) in 2015 [19]. Its prevalence among women was higher (26.4%) than that among men (22.8%) [19]. To recruit a sufficient number of participants for this study, 73 health facilities were selected from across Siaya county, which received more than 500 outpatients per month on average in 2015, based on records in the District Health Information System. Then, they were clustered into the six sub-counties (Ugenya, Ugunja, Alego-Usonga, Gem, Bondo, and Rarieda), and one facility was randomly selected per sub-county. Each of the six facilities was allocated a sample size proportional to the number of women of reproductive age who lived in each sub-county in 2009 [20].

### Participants

Study participants were sexually active women aged 18 to 49 who reported having had sexual intercourse at least once within 30 days prior to the interview. However, pregnant women and women who were sterilized were excluded because they were expected to have different reproductive characteristics from other women [14].

### Data collection

Data were collected through face-to-face interviews at the selected facilities from March to May 2017. For data collection, six experienced female research assistants were recruited. Before the data collection, a two-day training was conducted for the research assistants on data collection and ethical considerations. The research assistants approached women in an outpatient waiting area of the facilities and asked for their participation in the study. The first woman was selected purposively, and then every third woman that the research assistant saw in the outpatient waiting area was approached until the required sample size was obtained at each facility. Then, they conducted face-to-face interviews with the women who were willing to participate in the study using a structured questionnaire in a separate room in the facilities. Each interview lasted for about 30 minutes. All women received one bar soap as compensation for their participation.

### Questionnaire

A structured questionnaire was developed by adapting items from existing tools [17, 21, 22]. The questionnaire was first developed in English and then translated to Kiswahili. It was back-translated to English by a different researcher to ensure the accuracy of the translation.

### Measures

#### Consistent condom use

The outcome of this study was consistent condom use in the past 90 days. It was assessed with regular and non-regular partners, respectively. “Regular partner” was defined as either husbands or live-in sexual partners, and “non-regular partner” was defined as other partners with whom the woman had sexual intercourse in the past 90 days [21]. The frequency of condom use was asked with the following question: “With what frequency did you and all of your (regular or non-regular partners) use a male or female condom during the past 90 days?” Women answered this question using a four-point scale “every time,” “almost every time,” “sometimes,” and “never.” Only those who answered with “every time” were considered as having consistent condom use.

#### Highly effective contraceptive use

Women were classified into two categories based on their use of HECs in the past 90 days: HEC users and non-HEC users. HEC users were defined as those who reported using implants, injectable, intrauterine devices (IUDs), or contraceptive pills (OCPs). Non-HEC users were defined as those who did not report using any of these methods.

All women were asked if they were using any contraceptive methods with the following question: “Are you currently doing something or using any method to delay or avoid getting pregnant or to prevent HIV and sexually transmitted infections (STIs)?” Then, if the answer to this question was “yes,” they were asked which methods they had used in the past 90 days with an item: “Which methods have you used for the last 90 days?” Multiple answers were possible for this question.

#### Socio-demographic characteristics

Socio-demographic characteristics of women were included. They included the following: age, ethnicity, religion, education, employment, marital status, the number of children, and desire for having children in the future. Age and the number of children were collected as a continuous variable, and the others as a categorical variable. Age and the number of children were later categorized for analysis. Questions for socio-demographic characteristics were adopted from Kenya Demographic and Health Survey 2014 [17].

#### HIV status and perceived HIV risk

Women’s HIV status was asked via an item: “Have you ever been told by a health care provider that you have HIV?” [23]. ART status was also asked among HIV-positive women. Other statuses were asked such as if they are aware of their regular partner’s HIV status and vice versa, and the regular partner’s HIV status was also asked if they were aware. Non-regular partner’s HIV status was not obtained because some women can have two or more non-regular partners.

Perceived HIV risk was measured among women who reported their HIV status as negative or unknown. For this variable, the perceived risk of HIV infection scale (PRHS), an eight-item scale, was used [22]. Total scores of PRHS range from 10 to 40, where the higher scores indicate higher perceived risk [22]. For validation of PRHS in this study, scores of PRHS were compared with a single item measure of HIV risk perception, which was previously used in Kenya [24]. The total scores in this study ranged from 11 to 36 (Cronbach’s alpha 0.77).

#### HIV-related knowledge

HIV knowledge was measured by asking ten questions adopted from the Behavioral Surveillance Surveys (BSS) questionnaire [21]. Women were first asked if they had ever heard of a disease called HIV. If yes, the woman answered other nine questions about HIV transmission and prevention using “yes,” “no,” or “don’t know.” During the analysis, one point was given to each correct response. “Don’t know” was coded as an incorrect response. The knowledge score was obtained by summing the score of nine items. Thus, total scores range from zero to nine. A higher score means better knowledge of HIV. The score was zero if women replied they had never heard of HIV.

#### Risky sexual behaviours

Three risky sexual behaviours were measured: early sexual debut, multiple sexual partnership, and sex under the influence of alcohol or drugs. Early sexual debut is defined as having had first sexual intercourse at or before the age of 16 [25, 26]. All women were asked their age of sexual debut. The answers were dichotomized as early sexual debut or not. Multiple sexual partnership is defined as having two or more sexual partners [3, 27]. Multiple sexual partnership was assessed with numerical measures for the number of sexual partners in the past 90 days. The answers were dichotomized into having two or more sexual partners or not. Women were asked the frequency of sex under the influence of alcohol and drugs in the past 90 days [3, 8]. They answered this question using a four-point scale from “never” to “every time.” Women who did not answer “never” were considered as having sex under the influence of alcohol and drugs.

#### Partner’s attitude toward contraception

Regular partner’s attitude toward contraception was examined. Women were asked if they think their regular partner approves or disapproves of couples using a contraceptive method to avoid pregnancy [17].

#### Condom knowledge and accessibility

Condom accessibility was assessed by three questions [21]. First, all women were asked if they had heard of condoms. If yes, they were asked where they can get condoms with the response option of “don’t know”. Then, those who answered other than “don’t know” were asked how long it takes to obtain condoms from their home or workplace.

#### Reasons for not using condoms

An open-ended question was included to examine reasons for not using condoms. Women were asked why they did not use a condom during the last sexual intercourse.

### Data analysis

A total of 833 women completed the interview. This analysis excluded pregnant women (n = 79) and women who had been sterilized (n = 13). In addition, six women were excluded due to missing data. In total, data of 735 women were analyzed.

The women’s characteristics were summarized by using descriptive statistics. Chi-square or Fisher’s exact tests were used for categorical variables and independent sample t-tests for continuous variables. Multiple logistic regression was then performed to examine the associations between consistent condom use and each of the independent variables. The outcome was examined with regular and non-regular partners, separately. For condom use with a regular partner, the following variables were included: age, education, polygamous status, the history of unintended pregnancy, the number of children, pregnancy intention, women and their regular partner’s HIV status, HIV risk behaviours, condom accessibility, and partner’s attitude toward contraception. For condom use with a non-regular partner, the following variables were included: age, education, the history of unintended pregnancy, the number of children, women’s HIV status, pregnancy intention, HIV risk behaviours, and condom accessibility. In addition, a sub-sample analysis of HIV-negative or status unknown women was conducted. Moreover, multiple logistic regression was conducted to examine the association between the independent variables and dual-method use. Open-ended responses were analyzed to identify overarching themes by the principal researcher and three research assistants. Then, these results were compared among HEC users and non-users using Fisher’s exact test.

Statistical significance was set at a p-value of 0.05. Multicollinearity among independent variables was tested. Due to multicollinearity, the number of children was excluded from the sub-sample analysis and the multiple logistic regression of factors associated with dual-method use. Partner’s attitude toward contraception was also excluded from the sub-sample analysis. All data were coded and entered using EpiData version 3.1 and analyzed using Stata version 13.1.

### Ethical considerations

Ethical approval was obtained from the Research Ethics Committee of the Graduate School of Medicine, The University of Tokyo, Japan, the Kenyatta National Hospital and University of Nairobi Ethics and Research Committee, Kenya and National Commission for Science, Technology and Innovation, Kenya. Participation for this study was voluntary and written informed consent was taken before the interview with all women. To assure confidentiality, each interview was conducted in a private room in the health facilities, and participants’ names were not obtained.

## Results

Table 1 presents the socio-demographic characteristics of 735 women. The mean age was 28.4 (standard deviation [SD] 7.3) years. The majority (82.3%) were Luo ethnicity, and almost all (99.6%) were Christians. More than 60% had completed primary education. Out of 735 women, 655 (89.1%) were married or had been married, and 114 (15.5%) were in polygamous marriage. The majority (94.7%) had at least one child, and the mean number was 2.9 (SD 1.7). Many of the women (64.4%) had an intention of pregnancy in the future. Of all, 231 (31.4%) were HIV-positive, and 230 (31.3%) were on ART. Among 609 women who had a stable partner, 144 (23.7%) had HIV-positive concordant status and 49 (8.0%) had serodiscordant status, while 61 (10.0%) did not know their partner’s HIV status.

**Table 1:** Characteristics of women by HEC use and HIV status (n = 735).

Variables	HEC use /HIV- or unknown (n = 311)		Non-HEC use/HIV- or unknown (n = 193)		HEC use/HIV+ (n = 162)		Non-HEC use/HIV+ (n = 69)		Total (n = 735)		
	n	%	n	%	n	%	n	%	n	%	p-value^†^
**Age (range, 18–49; mean [SD], 28.4 [7.3])**											
18–24	140	45.0	90	46.6	30	18.5	20	29.0	280	38.1	**<0.001**
25–34	117	37.6	62	32.1	84	51.9	30	43.5	293	39.9	
35–49	54	17.4	41	21.2	48	29.6	19	27.5	162	22.0	
**Ethnicity**											
Luo	253	81.4	154	79.8	138	85.2	60	87.0	605	82.3	0.395
Others	58	18.7	39	20.2	24	14.8	9	13.0	130	17.7	
**Religion**											
Roman Catholic	62	19.9	53	27.5	30	18.5	15	21.7	160	21.8	0.115
Protestant/Other Christian	249	80.1	139	72.0	130	80.3	54	78.3	572	77.8	
Muslim	0	0	1	0.5	2	1.2	0	0	3	0.4	
**Education**											
Never	114	36.7	66	34.2	70	43.2	37	53.6	287	39.0	**0.006**
Primary	152	48.9	93	48.2	82	50.6	25	36.2	352	47.9	
Secondary	45	14.5	34	17.6	10	6.2	7	10.1	96	13.1	
**Employment status**											
Unemployed	60	19.3	42	21.8	20	12.4	9	13.0	131	17.8	**0.004**
Farmer	124	39.9	72	37.3	73	45.1	35	50.7	304	41.4	
Student	8	2.6	14	7.3	2	1.2	0	0	24	3.3	
Others	119	38.3	65	33.7	67	41.4	25	36.2	276	37.6	
**Marital status**											
Single	31	10.0	41	21.2	4	2.5	4	5.8	80	10.9	**<0.001**
Married/Divorced/Widow	280	90.0	152	78.8	158	97.5	65	94.2	655	89.1	
**Polygamous status**											
No/Don't know	264	84.9	171	88.6	129	79.6	57	82.6	621	84.5	0.131
Yes	47	15.1	22	11.4	33	20.4	12	17.4	114	15.5	
**No. of children (range, 0–8; mean [SD], 2.9 [1.7])**											
0	7	2.3	25	13.0	1	0.6	6	8.7	39	5.3	**<0.001**
1–2	142	45.7	86	44.6	53	32.7	25	36.2	306	41.6	
3+	162	52.1	82	42.5	108	66.7	38	55.1	390	53.1	
**Pregnancy intention**											
No/Don't know	109	35.1	50	25.9	78	48.2	25	36.2	262	35.7	**<0.001**
Yes	202	65.0	143	74.1	84	51.9	44	63.8	473	64.4	
**HIV sero-discordant/concordant relationship (n = 609)**^**1**^											
Both negative	242	89.0	113	78.5	0	0	0	0	355	58.3	
Both positive	-	-	-	-	107	77.5	37	67.3	144	23.7	
Respondent positive	-	-	-	-	17	12.3	13	23.6	30	4.9	
Partner positive	9	3.3	10	6.9	-	-	-	-	19	3.1	
Respondent negative and partner status unknown	21	7.7	21	14.6	-	-	-	-	42	6.9	
Respondent positive and partner status unknown	-	-	-	-	14	10.1	5	9.1	19	3.1	

HEC: highly effective contraceptive

^†^ Based on Chi-squared test except for religion which was analyzed with Fisher’s exact test

^1^Women who had a stable partner

Table 2 shows contraceptive methods used by women in the past 90 days before the interview. Out of 735 women, 473 (64.4%) reported having used HECs. Among HECs, implants were the most common method (32.0%) followed by injectable (27.1%), IUDs (3.4%), and OCPs (1.9%). Of all, 289 (39.3%) reported having used barrier methods in the past 90 days. Male condoms were the dominant barrier method (38.8%) compared to female condoms (0.8%). Compared to HIV-negative women or women with an unknown status, HIV-positive women were more likely to use HECs (70.1% vs. 61.7%, p = 0.027). They were also more likely to use barrier methods (61.9% vs. 29.0%, p<0.001).

**Table 2:** Contraceptive methods used by women in the past 90 days by HIV status^1^.

	HIV- or unknown (n = 504)		HIV+ (n = 231)		Total		p-value^†^
	n	%	n	%	n	%	
**Contraceptive use**	**397**	**78.8**	**204**	**88.3**	**601**	**81.8**	**0.002**
**Highly effective contraceptive use**	**311**	**61.7**	**162**	**70.1**	**473**	**64.4**	**0.027**
Implants	151	30.0	84	36.4	235	32.0	0.084
IUDs	12	2.4	13	5.6	25	3.4	**0.024**
Injectable	139	27.6	60	26.0	199	27.1	0.649
OCPs	9	1.8	5	2.2	14	1.9	0.727
**Barrier methods (n = 289)**^**2**^	**146**	**29.0**	**143**	**61.9**	**289**	**39.3**	**<0.001**
Male condom	144	28.6	141	61.0	285	38.8	**<0.001**
Female condom	3	0.6	3	1.3	6	0.8	0.325
**Traditional methods (n = 3)**							
Rhythm method	3	0.6	0	0.0	3	0.4	0.556

IUDs: intrauterine devices; OCPs: oral contraceptive pills

^†^ Based on Chi-squared test except for rhythm method which was analyzed with Fisher’s exact test

^1^ Multiple responses obtained.

^2^ Two women used both male and female condoms.

Table 3 illustrates condom use behaviors in sexual intercourse with regular and non-regular partners by HIV status. Out of 735 women, 595 (81.0%) had sexual intercourse with only their regular partner, 126 (17.1%) with only non-regular partners, and 14 (1.9%) with both types of partners in the 90 days before the interview.

**Table 3:** Condom use among women by HEC use and HIV status (n = 735).

Variables	HEC use /HIV- or unknown (n = 311)		Non-HEC use/HIV- or unknown (n = 193)		HEC use/HIV+ (n = 162)		Non-HEC use/HIV+ (n = 69)		Total (n = 735)		
	n	%	n	%	n	%	n	%	n	%	p-value^†^
**Used a condom every time with a regular partner in the past 90 days (n = 609)**											
No	258	94.9	118	81.9	88	63.8	29	52.7	493	81.0	**<0.001**
Yes	14	5.1	26	18.1	50	36.2	26	47.3	116	19.0	
**Used a condom every time with a non-regular partner in the past 90 days (n = 140)**											
No	34	79.1	23	45.1	17	54.8	6	40.0	80	57.1	**0.004**
Yes	9	20.9	28	54.9	14	45.2	9	60.0	60	42.9	

HEC: highly effective contraceptive

^†^ Based on Chi-squared test

Among 609 women who had sexual intercourse with their regular partner, 19.0% reported consistent condom use in the past 90 days. The proportion of women who reported consistent condom use was different by both HEC use and HIV status (p<0.001). Particularly among HEC users, HIV-positive women were more likely to use condoms consistently than HIV-negative or status unknown women (36.2% vs. 5.1%).

Of 140 women who had sexual intercourse with non-regular partners, 42.9% reported that they had used condoms consistently in the past 90 days. The proportion of women who reported consistent condom use was different by both HEC use and HIV status (p = 0.004). Particularly among HEC users, HIV-positive women were more likely to use condoms consistently than HIV-negative or status unknown women (45.2% vs. 20.9%).

Table 4 and Table 5 summarize reasons for not using a condom at the last sexual intercourse with regular and non-regular partners, respectively. Of 432 women who did not use a condom with a regular partner at the last sexual intercourse, 43.9% reported opposition from partners as a reason, 22.5% stated that it was because they trusted their partners, and 21.3% said that it was because they knew their partner’s HIV status or their partner was healthy. About one fifth (20.6%) stated it was because they used HECs. Among 51 women who did not use a condom with a non-regular partner at the last sexual intercourse, the common reasons for not using a condom were similar to those with regular partners: opposition from partners (35.3%), knowing their partner’s HIV status, or their partner was healthy (27.5%) and trusting their partners (21.2%). In total, 19 women expressed fear of side effects from using condoms. The major fears included that condoms can remain in the body and cause abdominal pain, cancer, and inflammation. Five women were afraid of suggesting condom use, as her partner might think she is cheating on him, a prostitute, or HIV-positive.

**Table 4:** Reasons why women did not use a condom at the last sexual intercourse with a regular partner (n = 432)^1^.

	HEC use /HIV- or unknown (n = 243)		Non-HEC use/HIV- or unknown (n = 103)		HEC use/HIV+ (n = 60)		Non-HEC use/HIV+ (n = 27)		Total (n = 432)		p-value^†^
	n	%	n	%	n	%	n	%	n	%	
Partner objected	90	37.0	44	42.7	44	73.3	12	44.4	190	43.9	**<0.001**
I trusted my partner/We were married	65	26.9	26	25.2	3	5.0	3	11.1	97	22.5	**<0.001**
I knew my partner's HIV status/My partner was healthy	68	28.1	18	17.5	5	8.3	1	3.7	92	21.3	**<0.001**
Used other contraceptives	74	30.6	0	0	15	25.0	0	0	89	20.6	<**0.001**
Don't like/Inconvenient	19	7.9	12	11.7	2	3.3	3	11.1	36	8.3	0.254
Didn't think of using a condom/I had never used a condom before	15	6.2	6	5.8	1	1.7	1	3.7	23	5.3	0.612
Not available/Too expensive	11	4.6	3	2.9	6	10.0	2	7.4	22	5.1	0.193
I wanted to get pregnancy	0	0	11	10.7	0	0	7	25.9	18	4.2	**<0.001**
Fear of side effects	10	4.1	3	2.9	3	5.0	2	7.4	18	4.2	0.606
I did not know how to suggest my partner to use a condom	1	0.4	3	2.9	1	1.7	0	0	5	1.2	0.160
I could not get pregnant	0	0	3	2.9	0	0	2	7.4	5	1.2	**0.002**
I did not know how to use a condom	1	0.4	2	1.9	0	0	0	0	3	0.7	0.357
No specific reasons/Don't know	4	1.7	4	3.9	0	0	0	0	8	1.9	0.344

HEC: highly effective contraceptive

^†^ Based on Fisher's exact test

^1^ Multiple responses obtained.

**Table 5:** Reasons why women did not use a condom at the last sexual intercourse with a non-regular partner (n = 51)^1^.

	HEC use /HIV- or unknown (n = 22)		Non-HEC use/HIV- or unknown (n = 14)		HEC use/HIV+ (n = 10)		Non-HEC use/HIV+ (n = 5)		Total (n = 51)		p-value^†^
	n	%	n	%	n	%	n	%	n	%	
Partner objected	6	27.3	3	21.4	8	80.0	1	20.0	18	35.3	**0.014**
I knew my partner's HIV status/My partner was healthy	9	40.9	5	35.7	0	0	0	0	14	27.5	**0.036**
I trusted my partner	5	22.7	4	26.7	1	10.0	1	20.0	11	21.2	0.885
Used other contraceptives	6	27.3	0	0	0	0	0	0	6	11.8	**0.049**
Not available/Too expensive	1	4.6	2	14.3	1	10.0	1	20.0	5	9.8	0.494
Don't like/Inconvenient	3	13.6	1	7.1	0	0	0	0	4	7.8	0.871
I wanted to get pregnancy	0	0	1	7.1	0	0	1	20.0	2	3.9	0.137
Fear of side effects	0	0	1	7.1	0	0	0	0	1	2.0	0.569
I could not get pregnant	0	0	1	7.1	0	0	0	0	1	2.0	0.569
No specific reasons/Don't know	1	4.6	0	0	0	0	1	20.0	2	3.9	0.295

HEC: highly effective contraceptive

^†^ Based on Fisher's exact test

^1^ Multiple responses obtained.

Table 6 demonstrates multiple logistic regression analyses of the factors associated with consistent condom use in the past 90 days with regular and non-regular partners, respectively. After adjusting for confounders, HEC use was significantly associated with decreased condom use with a regular partner (adjusted odds ratio (AOR) = 0.25; 95% CI 0.15–0.43, p<0.001) and with non-regular partners (AOR = 0.25; 95% CI 0.11–0.58, p = 0.001).

**Table 6:** Factors associated with consistent condom use in the past 90 days.

Variables	Consistent condom use with a regular partner (n = 609)						Consistent condom use with a non-regular partner (n = 140)					
	OR	95%CI	p	AOR ^1^	95%CI	p	OR	95%CI	p	AOR ^2^	95%CI	p
Non-HEC use	1			1			1			1		
HEC use	0.52	(0.26–0.49)	**0.002**	0.25	(0.15–0.43)	**<0.001**	0.35	(0.18–0.71)	**0.003**	0.25	(0.11–0.58)	**0.001**
**HIV status**												
Negative/Don't know				1						1		
Positive				4.16	(1.83–9.44)	**0.001**				3.42	(1.09–10.69)	**0.035**
**Partner's HIV status**												
Negative				1								
Positive				3.70	(1.51–9.02)	**0.004**						
Don't know				1.36	(0.55–3.35)	0.506						
**Partner’s attitude toward contraception**												
Disagree				1								
Agree/Don't know				12.56	(4.67–33.83)	**<0.001**						

OR: odds ratio, AOR: adjusted odds ratio; HEC: highly effective contraceptive

^1^ Adjusted for age, education, polygamous status, history of unintended pregnancy, number of children, pregnancy intention, HIV status, partner's HIV status, HIV-related knowledge, age of sexual debut, multiple sex partnership, sex under the influence of alcohol or drugs, condom accessibility, and partner's attitude toward contraception.

^2^ Adjusted for age, education, history of unintended pregnancy, number of children, pregnancy intention, HIV status, HIV-related knowledge, age of sexual debut, multiple sex partnership, sex under the influence of alcohol or drugs, and condom accessibility.

Among women with a regular partner, partner’s positive attitude toward contraception was significantly associated with increased condom use (AOR = 12.56; 95% CI 4.67–33.83, p<0.001). Compared with HIV-negative or status unknown women, HIV-positive women were more likely to use condoms consistently (AOR = 4.16; 95% CI 1.83–9.44, p = 0.001). Similarly, women with an HIV-positive partner were more likely to do so than those who had an HIV-negative or status unknown partner (AOR = 3.70; 95% CI 1.51–9.02, p = 0.004). Among women with a non-regular partner, their HIV-positive status was associated with consistent condom use (AOR = 3.42; 95% CI 1.09–10.69, p = 0.035).

Table 7 shows the results of sub-sample analyses among HIV-negative or status unknown women regarding the factors associated with condom use with regular and non-regular partners, respectively. The results showed similar associations between HEC use and condom use to those of the overall analyses (regular partners: AOR = 0.19; 95% CI 0.09–0.41, p<0.001; non-regular partners: AOR = 0.13; 95% CI 0.03–0.52, p = 0.004). Scores of PRHS were positively associated with consistent condom use with a regular partner (AOR = 1.10; 95% CI 1.02–1.18, p = 0.016). However, scores on PRHS were not significantly associated with consistent condom use with a non-regular partner (AOR = 0.91; 95% CI 0.82–1.02, p = 0.114).

**Table 7:** Factors associated with condom use among HIV-negative or status unknown women in the past 90 days.

Variables	Consistent condom use with a regular partner (n = 416)						Consistent condom use with a non-regular partner (n = 94)					
	OR	95%CI	p	AOR ^1^	95%CI	p	OR	95%CI	p	AOR ^2^	95%CI	p
Non-HEC use	1			1			1			1		
HEC use	0.25	(0.12–0.49)	**<0.001**	0.19	(0.09–0.41)	**<0.001**	0.22	(0.09–0.54)	**0.001**	0.13	(0.03–0.52)	**0.004**
**Wants more children**												
No				1						1		
Yes				0.60	(0.28–1.26)	0.175				9.69	(1.22–77.11)	**0.032**
**Perceived HIV risk**				1.10	(1.02–1.18)	**0.016**				0.91	(0.82–1.02)	0.114

OR: odds ratio; AOR: adjusted odds ratio; HEC: highly effective contraceptive

^1^ Adjusted for age, education, polygamous status, history of unintended pregnancy, pregnancy intention, HIV risk perception, HIV-related knowledge, age of sexual debut, multiple sex partnership, sex under the influence of alcohol or drugs, and condom accessibility. Number of children and partner’s attitude toward contraception were omitted because of multicollinearity.

^2^ Adjusted for age, education, history of unintended pregnancy, number of children, pregnancy intention, HIV risk perception, HIV-related knowledge, age of sexual debut, multiple sex partnership, sex under the influence of alcohol or drugs, and condom accessibility.

Table 8 demonstrates multiple logistic regression analyses of the factors associated with dual-method use in the past 90 days with regular and non-regular partners. HIV-positive status was significantly associated with dual-method use among women with a regular partner (AOR = 6.54; 95% CI 2.14–20.00, p = 0.001), but not among women with a non-regular partner (AOR = 2.90; 95% CI 0.90–9.38, p = 0.075). Among women with a regular partner, partner’s positive attitude toward contraception was significantly associated with dual-method use (AOR = 4.73; 95% CI 1.81–12.36, p = 0.002).

**Table 8:** Factors associated with dual-method use in the past 90 days.

Variables	Dual-method use with a regular partner (n = 609)						Dual-method use with a non-regular partner (n = 140)					
	OR	95%CI	p	AOR ^1^	95%CI	p	OR	95%CI	p	AOR ^2^	95%CI	p
**HIV status**												
Negative/Don't know	1			1			1			1		
Positive	10.04	(5.39–18.71)	**<0.001**	6.54	(2.14–20.00)	**0.001**	4.13	(1.63–10.48)	**0.003**	2.90	(0.90–9.38)	0.075
**Partner’s attitude toward contraception**												
Disagree				1								
Agree/Don't know			** **	4.73	(1.81–12.36)	**0.002**						

OR: odds ratio; AOR: adjusted odds ratio; HEC: highly effective contraceptive

1 Adjusted for age, education, polygamous status, the history of unintended pregnancy, pregnancy intention, HIV status, partner's HIV status, HIV-related knowledge, age of sexual debut, sex under the influence of alcohol or drugs, condom accessibility, and partner's attitude toward contraception. Number of children was omitted because of multicollinearity.

2 Adjusted for age, education, the history of unintended pregnancy, pregnancy intention, HIV status, HIV-related knowledge, age of sexual debut, multiple sex partnership, sex under the influence of alcohol or drugs, and condom accessibility. Number of children was omitted because of multicollinearity.

## Discussion

This study has two major findings. First, dual-method use was limited among HIV-negative women and women who had HIV-negative partners due to inconsistent condom use. Second, HEC use was negatively associated with consistent condom use, regardless of partner type and their HIV status.

HIV-positive women were more likely to practice dual-method use compared to HIV-negative or status unknown women. Pregnancy intention was lower among HIV-positive women than HIV-negative or status unknown women. A similar result was observed among Malawian women [28]. HIV-positive women may reduce their fertility intentions due to concerns of potential HIV transmission to infants [29], despite preventative interventions such as ART [30]. Lower childbearing desires can influence the utilization of HECs among HIV-positive women. HIV-positive status was also positively associated with condom use in Kenya and Malawi [31, 32]. This could be because HIV-positive women want to protect their HIV-negative partners from HIV infection. Women in seroconcordant HIV-positive relationships also may use a condom to prevent other STIs and HIV superinfection, which occurs when HIV-positive individuals interact with another HIV variant and causes an increase in viral load [33].

In contrast, dual-method use was relatively uncommon due to inconsistent condom use among HIV-negative women and women whose partner was HIV negative. Inconsistent condom use puts such women at a considerable risk of HIV infection from their partners. In sub-Saharan Africa, a great portion of HIV infections occur among HIV-serodiscordant couples [27]. Men tend to have more extramarital sex partners than women in sub-Saharan Africa [3, 27], and 44% of new HIV infections happened among married or cohabiting couples in Kenya in 2015 [13]. Among women who did not use a condom with a regular partner, the main reasons were the partner’s rejection and fidelity with their partners. This study also found that a regular partner’s positive attitude toward contraception was an important factor associated with condom use. However, in this study, HIV-negative or status unknown women perceiving themselves at higher risk for HIV were more likely to use condoms consistently with their regular partner. A similar association has been documented between perception of partner’s potential infidelity and consistent condom use among Latino youths in the USA [34]. This finding suggests that women are willing to use condoms when a higher risk is evident. Therefore, it is crucial to increase awareness about the risk of HIV infection from their intimate partners.

This study also found that HEC use can be a barrier to consistent condom use. It was positively associated with decreased condom use, regardless of partner type and their HIV status. This finding is consistent with results of previous studies in high-income settings [9, 10]. Women using HECs may be less likely to use condoms for three reasons. First, HEC users may no longer want to use condoms with their intimate partners. HECs provide better protection against unintended pregnancies than condoms [7, 35, 36]. Therefore, women may not use condoms if they think that their partners are healthy and faithful. In this study, HEC users were more likely to report the use of HECs as a reason for not using condoms with their regular partners than with non-regular partners. Second, women might hinder their ability to negotiate condom use with their intimate partners by using HECs. In sub-Saharan Africa, marital relations are generally regulated by fidelity and intimacy [37], and condoms are generally perceived as an HIV-preventive device used, not as a contraceptive method [38]. Condom use has been promoted by health staff and media to prevent HIV infection from high-risk partners [39, 40]. By using HECs, condoms are more likely to be perceived as a device for HIV prevention and stigmatized, and so condom use may become unacceptable, especially in marital sex. However, no statistically significant difference was observed in partner’s negative attitude about condom use between HEC and non-HEC users. Finally, although majority of women using HECs were aware that HECs could not prevent HIV infection, some believed that HECs could prevent HIV infection. Such women may stop using condoms when using HECs [41, 42].

The trade-off between HEC use and consistent condom use is a barrier to promoting dual protection, especially among HIV-negative women living in HIV-endemic settings. HECs are commonly used among women in a stable relationship, and such women are often at the considerable risk of HIV contraction from their partners [14]. Therefore, it is crucial to increase awareness about the risk of HIV infection from their partners and condom use, especially among women using HECs.

This study had several limitations. First, only women were included in this study. As condom use is not individual action, a couple-level analysis would be ideal [37]. Even though partner’s attitude toward contraceptive use was examined, it might have been misreported. Second, this study measured sexual behaviors, which is a sensitive subject. Therefore, behaviors may have been inaccurately reported. Especially, condom use could have been over-reported, and risky sexual behaviors under-reported. Nevertheless, reporting errors were minimized by assuring each woman of confidentiality of responses and conducting the interview in secured environments by experienced female interviewers. Third, as the data are cross-sectional, this study did not examine the causal inference of HECs on condom use. Fourth, due to the relatively small sample size of women who had sexual intercourse with a non-regular partner, some factors associated with condom use with non-regular partners might have been underestimated. Finally, the participants were recruited only at public health facilities. Thus, findings from this study may not be generalized to all women of reproductive age. Despite these limitations, this study has its value as it is the first study to document the association between the use of HECs and decreased condom use among HIV-infected and uninfected women in an HIV-endemic setting in Kenya.

## Conclusion

This study found dual-method use was limited, especially among HIV-negative women, and the use of HECs was significantly correlated with decreased condom use in Siaya county, Kenya. Due to this inverse association between HEC use and condom use, women may increase the risk of HIV infection from their sexual partners. The inverse association is also a significant barrier to promoting dual protection. The findings of this study should not deter scaling up HEC use but rather provide new insights into how to promote its use in sub-Saharan Africa. As dual-method use was insufficiently practiced among HIV-negative women, it should be promoted to protect them from HIV infection and unintended pregnancies. This study found that factors, such as partner’s positive attitude toward contraception and perceived HIV risk, may influence condom use among women. Therefore, family planning services should include a message about condom use and the possible risk of HIV infection from their intimate partners. Such a message should target not only women but also their partners, who may play a key role in condom use.

## Supporting information

S1 Table Factors associated with condom use with a regular partner among women (n = 609). (DOCX)

S2 Table Factors associated with condom use with a non-regular partner among women (n = 140). (DOCX)

S3 Table Factors associated with condom use with a regular partner among HIV-negative or status unknown women (n = 416). (DOCX)

S4 Table Factors associated with condom use with a non-regular partner among HIV-negative or status unknown women (n = 94). (DOCX)

S5 Table Factors associated with dual-method use with a regular partner (n = 609). (DOCX)

S6 Table Factors associated with dual-method use with a non-regular partner (n = 140). (DOCX)

S1 File Questionnaire (English). (PDF)

S2 File Questionnaire (Kiswahili). (PDF)
