# Supplementary material for: Consistent condom use among highly effective contraceptive users in an HIV-endemic area in rural Kenya
Source: PLoS One. 2019 May 6;14(5):e0216208. doi: 10.1371/journal.pone.0216208 (PMC6502455; doi:10.1371/journal.pone.0216208)
Supplement: S1 Table — (DOCX) [file pone.0216208.s001.docx]

**S1 Table. Factors associated with condom use with a regular partner among women (n=609)**

| **Variables** | **Consistent condom use with a regular partner in the past 90 days** | | | | | | |
| --- | --- | --- | --- | --- | --- | --- | --- |
|  | **OR** | **95%CI** | **p** |  | **AOR ^1^** | **95%CI** | **p** |
| **Contraceptive type** | |  |  |  |  |  |  |
| Non-HEC use | 1.00 |  |  |  | 1.00 |  |  |
| HEC use | 0.52 | (0.26-0.49) | **0.002** |  | 0.25 | (0.15-0.43) | **<0.001** |
|  |  |  |  |  |  |  |  |
| **1)Socio-demographic characteristics** | | |  |  |  |  |  |
| **Age** |  |  |  |  |  |  |  |
| 18-24 |  |  |  |  | 1.00 |  |  |
| 25-34 |  |  |  |  | 0.63 | (0.30-1.33) | 0.225 |
| 35-49 |  |  |  |  | 1.31 | (0.58-2.95) | 0.514 |
| **Education** |  |  |  |  |  |  |  |
| Never |  |  |  |  | 1.00 |  |  |
| Primary |  |  |  |  | 0.80 | (0.46-1.39) | 0.429 |
| Secondary |  |  |  |  | 1.96 | (0.79-4.83) | 0.145 |
| **Polygamous status** | |  |  |  |  |  |  |
| No/Don't know |  |  |  |  | 1.00 |  |  |
| Yes |  |  |  |  | 1.33 | (0.70-2.51) | 0.379 |
| **Had an unintended pregnancy** | | |  |  |  |  |  |
| No |  |  |  |  | 1.00 |  |  |
| Yes |  |  |  |  | 0.96 | (0.58-1.61) | 0.889 |
| **No. of children** | |  |  |  |  |  |  |
| 0 |  |  |  |  | 1.00 |  |  |
| 1-2 |  |  |  |  | 1.67 | (0.26-10.77) | 0.588 |
| 3+ |  |  |  |  | 2.06 | (0.30-14.05) | 0.460 |
| **Wants more children** | |  |  |  |  |  |  |
| No |  |  |  |  | 1.00 |  |  |
| Yes |  |  |  |  | 0.83 | (0.47-1.49) | 0.539 |
|  |  |  |  |  |  |  |  |
|  |  |  |  |  |  |  |  |
| **2) HIV status** | |  |  |  |  |  |  |
| **HIV status** |  |  |  |  |  |  |  |
| Negative/Don't know |  |  |  |  | 1.00 |  |  |
| Positive |  |  |  |  | 4.16 | (1.83-9.44) | **0.001** |
| **Partner's HIV status** | |  |  |  |  |  |  |
| Negative |  |  |  |  | 1.00 |  |  |
| Positive |  |  |  |  | 3.70 | (1.51-9.02) | **0.004** |
| Don't know |  |  |  |  | 1.36 | (0.55-3.35) | 0.506 |
|  |  |  |  |  |  |  |  |
| **3) HIV knowledge score** |  |  |  |  | 1.02 | (0.83-1.24) | 0.869 |
|  |  |  |  |  |  |  |  |
| **4)Risky sexual behaviors** | | |  |  |  |  |  |
| **Age of sexual debut** | |  |  |  |  |  |  |
| ≦15 years old |  |  |  |  | 1.00 |  |  |
| >16 years old |  |  |  |  | 1.30 | (0.78-2.15) | 0.317 |
| Don't know/No response |  |  |  |  | 0.55 | (0.07-4.52) | 0.576 |
| **Had multiple sex partners in the past 90 days** | | | |  |  |  |  |
| No |  |  |  |  | 1.00 |  |  |
| Yes |  |  |  |  | 0.20 | (0.02-2.20) | 0.189 |
| **Drank alcohol or used drugs before sex in the past 90 days** | | | | | |  |  |
| No |  |  |  |  | 1.00 |  |  |
| Yes |  |  |  |  | 1.26 | (0.62-2.54) | 0.520 |
|  |  |  |  |  |  |  |  |
|  |  |  |  |  |  |  |  |
| **5) Psychosocial characteristics about contraception** | | | | |  |  |  |
| **Necessary time to obtain condoms** | | |  |  |  |  |  |
| Under 1 hour |  |  |  |  | 1.00 |  |  |
| More than 1 hour |  |  |  |  | 1.62 | (0.98-2.69) | 0.062 |
| **Partner’s attitude toward contraception** | | | |  |  |  |  |
| Disagree |  |  |  |  | 1.00 |  |  |
| Agree/Don't know |  |  |  |  | 12.56 | (4.67-33.83) | **<0.001** |

OR: odds ratio; AOR: adjusted odds ratio; HEC: highly effective contraceptive

^1^ Adjusted for age, education, polygamous status, history of unintended pregnancy, number of children, pregnancy intention, HIV status, partner's HIV status, HIV-related knowledge, age of sexual debut, multiple sex partnership, sex under the influence of alcohol or drugs, condom accessibility, and partner's attitude toward contraception.
