# Supplementary material for: Consistent condom use among highly effective contraceptive users in an HIV-endemic area in rural Kenya
Source: PLoS One. 2019 May 6;14(5):e0216208. doi: 10.1371/journal.pone.0216208 (PMC6502455; doi:10.1371/journal.pone.0216208)
Supplement: S2 Table — (DOCX) [file pone.0216208.s002.docx]

**S2 Table. Factors associated with condom use with a non-regular partner among women (n=140)**

| **Variables** | **Consistent condom use with a non-regular partner in the past 90 days** | | | | | | |
| --- | --- | --- | --- | --- | --- | --- | --- |
|  | **OR** | **95%CI** | **p** |  | **AOR ^1^** | **95%CI** | **p** |
| **Contraceptive type** | |  |  |  |  |  |  |
| Non-HEC use | 1.00 |  |  |  | 1.00 |  |  |
| HEC use | 0.35 | (0.18-0.71) | **0.003** |  | 0.25 | (0.11-0.58) | **0.001** |
|  |  |  |  |  |  |  |  |
| **1)Socio-demographic characteristics** | | |  |  |  |  |  |
| **Age** |  |  |  |  |  |  |  |
| 18-24 |  |  |  |  | 1.00 |  |  |
| 25-34 |  |  |  |  | 1.23 | (0.36-4.20) | 0.739 |
| 35-49 |  |  |  |  | 0.75 | (0.17-3.27) | 0.699 |
| **Education** |  |  |  |  |  |  |  |
| Never |  |  |  |  | 1.00 |  |  |
| Primary |  |  |  |  | 1.64 | (0.63-4.23) | 0.308 |
| Secondary or more |  |  |  |  | 1.01 | (0.31-3.35) | 0.983 |
| **Had an unintended pregnancy** | | |  |  |  |  |  |
| No |  |  |  |  | 1.00 |  |  |
| Yes |  |  |  |  | 0.83 | (0.31-2.25) | 0.715 |
| **No. of children** | |  |  |  |  |  |  |
| 0 |  |  |  |  |  |  |  |
| 1-2 |  |  |  |  | 1.10 | (0.32-3.79) | 0.878 |
| 3+ |  |  |  |  | 1.20 | (0.19-7.83) | 0.846 |
| **Wants more children** | |  |  |  |  |  |  |
| No |  |  |  |  | 1.00 |  |  |
| Yes |  |  |  |  | 2.14 | (0.78-5.88) | 0.139 |
|  |  |  |  |  |  |  |  |
| **2) HIV status** | |  |  |  |  |  |  |
| **HIV status** |  |  |  |  |  |  |  |
| Negative/Don't know |  |  |  |  | 1.00 |  |  |
| Positive |  |  |  |  | 3.42 | (1.09-10.69) | **0.035** |
|  |  |  |  |  |  |  |  |
| **3) HIV knowledge score** |  |  |  |  | 1.38 | (0.94-2.03) | 0.103 |
|  |  |  |  |  |  |  |  |
| **4)** **Risky sexual behaviors** | | |  |  |  |  |  |
| **Age of sexual debut** | |  |  |  |  |  |  |
| ≦15 years old |  |  |  |  | 1.00 |  |  |
| >16 years old |  |  |  |  | 0.72 | (0.33-1.60) | 0.424 |
| **Had multiple sex partners in the past 90 days** | | | |  |  |  |  |
| No |  |  |  |  | 1.00 |  |  |
| Yes |  |  |  |  | 1.04 | (0.37-2.92) | 0.941 |
| **Drank alcohol or used drugs before sex in the past 90 days** | | | | | |  |  |
| No |  |  |  |  | 1.00 |  |  |
| Yes |  |  |  |  | 0.56 | (0.18-1.78) | 0.323 |
|  |  |  |  |  |  |  |  |
| **5) Psychosocial characteristics about contraception** | | | | |  |  |  |
| **Necessary time to obtain condoms** | | |  |  |  |  |  |
| Under 1 hour |  |  |  |  | 1.00 |  |  |
| More than 1 hour |  |  |  |  | 2.30 | (0.97-5.46) | 0.060 |

OR: odds ratio; AOR: adjusted odds ratio; HEC: highly effective contraceptive

^1^ Adjusted for age, education, history of unintended pregnancy, number of children, pregnancy intention, HIV status, HIV-related knowledge, age of sexual debut, multiple sex partnership, sex under the influence of alcohol or drugs, and condom accessibility.
