# Supplementary material for: Consistent condom use among highly effective contraceptive users in an HIV-endemic area in rural Kenya
Source: PLoS One. 2019 May 6;14(5):e0216208. doi: 10.1371/journal.pone.0216208 (PMC6502455; doi:10.1371/journal.pone.0216208)
Supplement: S3 Table — (DOCX) [file pone.0216208.s003.docx]

**S3 Table. Factors associated with condom use with a regular partner among HIV-negative or unknown women (n=416)**

| **Variables** | **Consistent condom use with a regular partner in the past 90 days** | | | | | | |
| --- | --- | --- | --- | --- | --- | --- | --- |
|  | **OR** | **95%CI** | **p** |  | **AOR^1^** | **95%CI** | **p** |
| **Contraceptive type** | |  |  |  |  |  |  |
| Non-HEC use | 1.00 |  |  |  | 1.00 |  |  |
| HEC use | 0.25 | (0.12-0.49) | **<0.001** |  | 0.19 | (0.09-0.41) | **<0.001** |
|  |  |  |  |  |  |  |  |
|  |  |  |  |  |  |  |  |
| **1)Socio-demographic characteristics** | | |  |  |  |  |  |
| **Age** |  |  |  |  |  |  |  |
| 18-24 |  |  |  |  | 1.00 |  |  |
| 25-34 |  |  |  |  | 0.76 | (0.33-1.75) | 0.526 |
| 35-49 |  |  |  |  | 1.09 | (0.40-2.98) | 0.864 |
| **Education** |  |  |  |  |  |  |  |
| Never |  |  |  |  | 1.00 |  |  |
| Primary |  |  |  |  | 0.56 | (0.26-1.21) | 0.140 |
| Secondary or more |  |  |  |  | 0.69 | (0.22-2.21) | 0.537 |
| **Polygamous status** | |  |  |  |  |  |  |
| No/Don't know |  |  |  |  | 1.00 |  |  |
| Yes |  |  |  |  | 0.88 | (0.32-2.45) | 0.810 |
| **Had an unintended pregnancy** | | |  |  |  |  |  |
| No |  |  |  |  | 1.00 |  |  |
| Yes |  |  |  |  | 1.04 | (0.51-2.14) | 0.913 |
| **No. of children^2^** | |  |  |  |  |  |  |
| 0 |  |  |  |  |  |  |  |
| 1-2 |  |  |  |  |  |  |  |
| 3+ |  |  |  |  |  |  |  |
| **Wants more children** | |  |  |  |  |  |  |
| No |  |  |  |  | 1.00 |  |  |
| Yes |  |  |  |  | 0.60 | (0.28-1.26) | 0.175 |
|  |  |  |  |  |  |  |  |
|  |  |  |  |  |  |  |  |
| **2) Perceived HIV risk** |  |  |  |  | 1.10 | (1.02-1.18) | **0.016** |
|  |  |  |  |  |  |  |  |
|  |  |  |  |  |  |  |  |
| **3) HIV knowledge score** |  |  |  |  | 1.16 | (0.86-1.57) | 0.342 |
|  |  |  |  |  |  |  |  |
|  |  |  |  |  |  |  |  |
| **4) Risky sexual behaviors** | | |  |  |  |  |  |
| **Age of sexual debut** | |  |  |  |  |  |  |
| ≦15 years old |  |  |  |  | 1.00 |  |  |
| >16 years old |  |  |  |  | 1.85 | (0.88-3.89) | 0.104 |
| **Had multiple sex partners in the past 90 days** | | | |  |  |  |  |
| No |  |  |  |  | 1.00 |  |  |
| Yes |  |  |  |  | 2.73 | (0.25-29.56) | 0.410 |
| **Drank alcohol or used drugs before sex in the past 90 days** | | | | | |  |  |
| No |  |  |  |  | 1.00 |  |  |
| Yes |  |  |  |  | 0.35 | (0.12-1.03) | 0.056 |
|  |  |  |  |  |  |  |  |
| **5) Psychosocial characteristics about contraception** | | | | |  |  |  |
| **Necessary time to obtain condoms** | | |  |  |  |  |  |
| Under 1 hour |  |  |  |  | 1.00 |  |  |
| More than 1 hour |  |  |  |  | 1.37 | (0.65-2.88) | 0.412 |
| **Partner’s attitude toward contraception^2^** | | | |  |  |  |  |
| Disagree |  |  |  |  |  |  |  |
| Agree/Don't know |  |  |  |  |  |  |  |

OR: odds ratio; AOR: adjusted odds ratio; HEC: highly effective contraceptive

^1^ Adjusted for age, education, polygamous status, history of unintended pregnancy, pregnancy intention, HIV risk perception, HIV-related knowledge, age of sexual debut, multiple sex partnership, sex under the influence of alcohol or drugs, condom accessibility, and partner's attitude toward contraception.

^2^ Number of children and partner’s attitude toward contraception were omitted because of multicollinearity.
