# Supplementary material for: Consistent condom use among highly effective contraceptive users in an HIV-endemic area in rural Kenya
Source: PLoS One. 2019 May 6;14(5):e0216208. doi: 10.1371/journal.pone.0216208 (PMC6502455; doi:10.1371/journal.pone.0216208)
Supplement: S4 Table — (DOCX) [file pone.0216208.s004.docx]

**S4 Table. Factors associated with condom use with a non-regular partner among HIV-negative or status unknown women (n=94)**

| **Variables** | **Consistent condom use with a non-regular partner in the past 90 days** | | | | | | |
| --- | --- | --- | --- | --- | --- | --- | --- |
|  | **OR** | **95%CI** | **p** |  | **AOR^1^** | **95%CI** | **p** |
| **Contraceptive type** | |  |  |  |  |  |  |
| Non-HEC use | 1.00 |  |  |  | 1.00 |  |  |
| HEC use | 0.22 | (0.09-0.54) | **0.001** |  | 0.13 | (0.03-0.52) | **0.004** |
|  |  |  |  |  |  |  |  |
| **1)Socio-demographic characteristics** | | |  |  |  |  |  |
| **Age** |  |  |  |  |  |  |  |
| 18-24 |  |  |  |  | 1.00 |  |  |
| 25-34 |  |  |  |  | 4.92 | (0.87-27.93) | 0.072 |
| 35-49 |  |  |  |  | 0.08 | (0.00-1.48) | 0.090 |
| **Education** |  |  |  |  |  |  |  |
| Never |  |  |  |  | 1.00 |  |  |
| Primary |  |  |  |  | 2.75 | (0.52-14.60) | 0.234 |
| Secondary or more |  |  |  |  | 1.56 | (0.29-8.49) | 0.605 |
| **Had an unintended pregnancy** | | |  |  |  |  |  |
| No |  |  |  |  | 1.00 |  |  |
| Yes |  |  |  |  | 0.51 | (0.11-2.46) | 0.401 |
| **No. of children** | |  |  |  |  |  |  |
| 0 |  |  |  |  | 1.00 |  |  |
| 1-2 |  |  |  |  | 1.59 | (0.30-8.54) | 0.590 |
| 3+ |  |  |  |  | 2.65 | (0.13-53.90) | 0.525 |
| **Wants more children** | |  |  |  |  |  |  |
| No |  |  |  |  | 1.00 |  |  |
| Yes |  |  |  |  | 9.69 | (1.22-77.11) | **0.032** |
|  |  |  |  |  |  |  |  |
| **2) Perceived HIV risk** |  |  |  |  | 0.91 | (0.82-1.02) | 0.114 |
|  |  |  |  |  |  |  |  |
| **3) HIV knowledge score** |  |  |  |  | 2.02 | (0.96-4.23) | 0.063 |
|  |  |  |  |  |  |  |  |
| **4) Risky sexual behaviors** | | |  |  |  |  |  |
| **Age of sexual debut** | |  |  |  |  |  |  |
| ≦15 years old |  |  |  |  | 1.00 |  |  |
| >16 years old |  |  |  |  | 1.21 | (0.35-4.17) | 0.759 |
| **Had multiple sex partners in the past 90 days** | | | |  |  |  |  |
| No |  |  |  |  | 1.00 |  |  |
| Yes |  |  |  |  | 0.72 | (0.16-3.21) | 0.668 |
| **Drank alcohol or used drugs before sex in the past 90 days** | | | | | |  |  |
| No |  |  |  |  | 1.00 |  |  |
| Yes |  |  |  |  | 0.11 | (0.01-1.47) | 0.096 |
|  |  |  |  |  |  |  |  |
| **5) Psychosocial characteristics about contraception** | | | | |  |  |  |
| **Necessary time to obtain condoms** | | |  |  |  |  |  |
| Under 1 hour |  |  |  |  | 1.00 |  |  |
| More than 1hour |  |  |  |  | 3.57 | (0.75-16.93) | 0.109 |

OR: odds ratio; AOR: adjusted odds ratio; HEC: highly effective contraceptive

^1^Adjusted for age, education, history of unintended pregnancy, number of children, pregnancy intention, HIV risk perception, HIV-related knowledge, age of sexual debut, multiple sex partnership, sex under the influence of alcohol or drugs, and condom accessibility.
