# Supplementary material for: Consistent condom use among highly effective contraceptive users in an HIV-endemic area in rural Kenya
Source: PLoS One. 2019 May 6;14(5):e0216208. doi: 10.1371/journal.pone.0216208 (PMC6502455; doi:10.1371/journal.pone.0216208)
Supplement: S5 Table — (DOCX) [file pone.0216208.s005.docx]

**S5 Table. Factors associated with dual-method use with a regular partner (n=609)**

| **Variables** | **Dual-method use with a regular partner in the past 90 days** | | | | | | |
| --- | --- | --- | --- | --- | --- | --- | --- |
|  | **OR** | **95%CI** | **p** |  | **AOR^1^** | **95%CI** | **p** |
| **1)Socio-demographic characteristics** | | |  |  |  |  |  |
| **Age** |  |  |  |  |  |  |  |
| 18-24 |  |  |  |  | 1.00 |  |  |
| 25-34 |  |  |  |  | 0.67 | (0.31-1.43) | 0.303 |
| 35-49 |  |  |  |  | 1.28 | (0.52-3.17) | 0.590 |
| **Education** |  |  |  |  |  |  |  |
| Never |  |  |  |  | 1.00 |  |  |
| Primary |  |  |  |  | 0.77 | (0.41-1.44) | 0.409 |
| Secondary or more |  |  |  |  | 0.63 | (0.19-2.10) | 0.451 |
| **Polygamous status** | |  |  |  |  |  |  |
| No/Don't know |  |  |  |  | 1.00 |  |  |
| Yes |  |  |  |  | 1.39 | (0.68-2.83) | 0.365 |
| **Had an unintended pregnancy** | | |  |  |  |  |  |
| No |  |  |  |  | 1.00 |  |  |
| Yes |  |  |  |  | 1.23 | (0.67-2.26) | 0.496 |
| **No. of children^2^** | |  |  |  |  |  |  |
| 0 |  |  |  |  |  |  |  |
| 1-2 |  |  |  |  |  |  |  |
| 3+ |  |  |  |  |  |  |  |
| **Wants more children** | |  |  |  |  |  |  |
| No |  |  |  |  | 1.00 |  |  |
| Yes |  |  |  |  | 0.79 | (0.41-1.55) | 0.499 |
|  |  |  |  |  |  |  |  |
|  |  |  |  |  |  |  |  |
| **2) HIV status** | |  |  |  |  |  |  |
| **HIV status** |  |  |  |  |  |  |  |
| Negative/Don't know | 1.00 |  |  |  | 1.00 |  |  |
| Positive | 10.04 | (5.39-18.71) | **<0.001** |  | 6.54 | (2.14-20.00) | **0.001** |
| **Partner’s HIV status** | |  |  |  |  |  |  |
| Negative |  |  |  |  | 1.00 |  |  |
| Positive |  |  |  |  | 2.03 | (0.67-6.15) | 0.212 |
| Don't know |  |  |  |  | 0.45 | (0.08-2.53) | 0.368 |
|  |  |  |  |  |  |  |  |
| **3) HIV knowledge score** |  |  |  |  | 0.95 | (0.74-1.23) | 0.723 |
|  |  |  |  |  |  |  |  |
|  |  |  |  |  |  |  |  |
| **4) Risky sexual behaviors** | | |  |  |  |  |  |
| **Age of sexual debut** | |  |  |  |  |  |  |
| ≦15 years old |  |  |  |  | 1.00 |  |  |
| >16 years old |  |  |  |  | 1.51 | (0.82-2.79) | 0.184 |
| Don't know/No response |  |  |  |  | 1.33 | (0.17-10.38) | 0.788 |
| **Had multiple sex partners in the past 90 days^3^** | | | |  |  |  |  |
| No |  |  |  |  |  |  |  |
| Yes |  |  |  |  |  |  |  |
| **Drank alcohol or used drugs before sex in the past 90 days** | | | | | |  |  |
| No |  |  |  |  | 1.00 |  |  |
| Yes |  |  |  |  | 0.97 | (0.44-2.16) | 0.945 |
|  |  |  |  |  |  |  |  |
| **5) Psychosocial characteristics about contraception** | | | | |  |  |  |
| **Necessary time to obtain condoms** | | |  |  |  |  |  |
| Under 1 hour |  |  |  |  | 1.00 |  |  |
| More than 1 hour |  |  |  |  | 1.42 | (0.78-2.58) | 0.249 |
| **Partner’s attitude toward contraception** | | | |  |  |  |  |
| Disagree |  |  |  |  | 1.00 |  |  |
| Agree/Don't know |  |  |  |  | 4.73 | (1.81-12.36) | **0.002** |

OR: odds ratio; AOR: adjusted odds ratio

^1^ Adjusted for age, education, polygamous status, history of unintended pregnancy, pregnancy intention, HIV status, partner's HIV status, HIV-related knowledge, age of sexual debut, sex under the influence of alcohol or drugs, condom accessibility, and partner's attitude toward contraception.

^2^ Number of children was omitted because of multicollinearity.

^3^ Multiple sex partnership predicted failure perfectly.
