# Supplementary material for: Consistent condom use among highly effective contraceptive users in an HIV-endemic area in rural Kenya
Source: PLoS One. 2019 May 6;14(5):e0216208. doi: 10.1371/journal.pone.0216208 (PMC6502455; doi:10.1371/journal.pone.0216208)
Supplement: S6 Table — (DOCX) [file pone.0216208.s006.docx]

**S6 Table. Factors associated with dual-method use with a non-regular partner (n=140)**

| **Variables** | **Dual-method use with a non-regular partner in the past 90 days** | | | | | | |
| --- | --- | --- | --- | --- | --- | --- | --- |
|  | **OR** | **95%CI** | **p** |  | **AOR^1^** | **95%CI** | **p** |
| **1)Socio-demographic characteristics** | | |  |  |  |  |  |
| **Age** |  |  |  |  |  |  |  |
| 18-24 |  |  |  |  | 1.00 |  |  |
| 25-34 |  |  |  |  | 1.87 | (0.44-7.99) | 0.396 |
| 35-49 |  |  |  |  | 1.61 | (0.46-5.67) | 0.458 |
| **Education** |  |  |  |  |  |  |  |
| Never |  |  |  |  | 1.00 |  |  |
| Primary |  |  |  |  | 1.11 | (0.37-3.30) | 0.409 |
| Secondary or more |  |  |  |  | 0.42 | (0.70-2.53) | 0.344 |
| **Had an unintended pregnancy** | | |  |  |  |  |  |
| No |  |  |  |  | 1.00 |  |  |
| Yes |  |  |  |  | 0.46 | (0.15-1.41) | 0.174 |
| **No. of children^2^** | |  |  |  |  |  |  |
| 0 |  |  |  |  |  |  |  |
| 1-2 |  |  |  |  |  |  |  |
| 3+ |  |  |  |  |  |  |  |
| **Wants more children** | |  |  |  |  |  |  |
| No |  |  |  |  | 1.00 |  |  |
| Yes |  |  |  |  | 0.86 | (0.30-2.49) | 0.783 |
|  |  |  |  |  |  |  |  |
|  |  |  |  |  |  |  |  |
| **2) HIV status** | |  |  |  |  |  |  |
| **HIV status** |  |  |  |  |  |  |  |
| Negative/Don't know | 1.00 |  |  |  | 1.00 |  |  |
| Positive | 4.13 | (1.63-10.48) | **0.003** |  | 2.90 | (0.90-9.38) | 0.075 |
|  |  |  |  |  |  |  |  |
| **3) HIV knowledge score** |  |  |  |  | 1.23 | (0.77-1.96) | 0.384 |
|  |  |  |  |  |  |  |  |
|  |  |  |  |  |  |  |  |
| **4) Risky sexual behaviors** | | |  |  |  |  |  |
| **Age of sexual debut** | |  |  |  |  |  |  |
| ≦15 years old |  |  |  |  | 1.00 |  |  |
| >16 years old |  |  |  |  | 0.49 | (0.16-1.49) | 0.208 |
| **Had multiple sex partners in the past 90 days** | | | |  |  |  |  |
| No |  |  |  |  | 1.00 |  |  |
| Yes |  |  |  |  | 1.59 | (0.53-4.77) | 0.404 |
| **Drank alcohol or used drugs before sex in the past 90 days** | | | | | |  |  |
| No |  |  |  |  | 1.00 |  |  |
| Yes |  |  |  |  | 1.67 | (0.57-4.89) | 0.352 |
|  |  |  |  |  |  |  |  |
| **5) Psychosocial characteristics about contraception** | | | | |  |  |  |
| **Necessary time to obtain condoms** | | |  |  |  |  |  |
| Under 1 hour |  |  |  |  | 1.00 |  |  |
| More than 1 hour |  |  |  |  | 1.36 | (0.47-3.92) | 0.572 |

OR: odds ratio; AOR: adjusted odds ratio

^1^ Adjusted for age, education, history of unintended pregnancy, pregnancy intention, HIV status, HIV-related knowledge, age of sexual debut, multiple sex partnership, sex under the influence of alcohol or drugs, and condom accessibility.

^2^ Number of children was omitted because of multicollinearity.
