# Supplementary material for: Consistent condom use among highly effective contraceptive users in an HIV-endemic area in rural Kenya
Source: PLoS One. 2019 May 6;14(5):e0216208. doi: 10.1371/journal.pone.0216208 (PMC6502455; doi:10.1371/journal.pone.0216208)
Supplement: S1 File — (PDF) [file pone.0216208.s007.pdf]

NO. □-□□□

Date:	D D / M M / Y Y Y Y
Facility:	
Interviewer:	
Field editor:	

QUESTIONAIRE

A) BACKGROUND INFORMATION

	Questions	Responses	Skip
A-1	How old are you ? (Completed year)	Age in completed years............................ □□<br>□88 Don't know	
A-2	What is your ethnicity?	□1 Luo<br>□2 Kisii<br>□3 Luhya<br>□4 Kalenjin<br>□5 Kikuyu<br>□6 Other ( )	
A-3	What is your religion?	□1 Roman catholic<br>□2 Protestant/other Christian<br>□3 Muslim<br>□4 No religion<br>□5 Other ( )	
A-4	What is the highest level of school you have attended?	□0 Never<br>□1 Incomplete primary school<br>□2 Complete primary school<br>□3 Incomplete secondary school<br>□4 Complete secondary school<br>□5 Higher than secondary school	
A-5	What is your occupation?	□0 Unemployed<br>□1 Agriculture<br>□2 Government worker<br>□3 Private sector<br>□4 Own business<br>□5 Students<br>□6 Other ( )	
A-6	Does your household have:<br>a) Electricity ?<br>b) A radio?<br>c) A television?<br>d) A mobile phone?<br>e) A non-mobile phone?<br>f) A refrigerator?<br>g) A solar panel?<br>h) A table?<br>i) A chair?<br>j) A sofa?<br>k) A bed?<br>l) A cupboard?<br>m) A clock?<br>n) A microwave oven?<br>o) A DVD player?<br>p) A cassetle or CD player?<br>q) A car or truck?<br>r) A bicycle?	NO / YES<br>a) Electricity □0 □1<br>b) Radio □0 □1<br>c) Television □0 □1<br>d) Mobile phone □0 □1<br>e) Non-mobile phone □0 □1<br>f) Refrigerator □0 □1<br>g) Solar panel □0 □1<br>h) Table □0 □1<br>i) Chair □0 □1<br>j) Sofa □0 □1<br>k) Bed □0 □1<br>l) Cupboard □0 □1<br>m) Clock □0 □1<br>n) Microwave oven □0 □1<br>o) DVD player □0 □1<br>p) Cassetle or CD player □0 □1<br>q) Car or truck □0 □1<br>r) Bicycle □0 □1	
A-7	What is your marital status?	□1 Single<br>□2 Married<br>□3 Widow<br>□4 Divorced	
A-8	Are you **currently** married or living with a man with whom you have a sexual relationship?	□1 Currently married, living with spouse<br>□2 Currently married, living with other sexual partner<br>□3 Currently married, not living with spouse or any other sexual partner<br>□4 Not married, living with sexual partner<br>□5 Not married, not living with sexual partner<br>□99 No response	 <br><br>→A-10<br>→A-10

A-9	Does your husband have other wives?	☐0 No<br>☐1 Yes<br>☐88 Don't know	
A-10	Are you pregnant now?	☐0 No<br>☐1 Yes<br>☐2 Unsure	
A-11	Have you ever given birth?	☐0 No<br>☐1 Yes	→A-14
A-12	How many chidren have you ever delivered?<br>IF NONE, RECORD '00'.	.... ☐☐	
A-13	How many living children do you have?<br>IF NONE, RECORD '00'.	.... ☐☐	
A-14	Would you like to have (a/another) child, or would you prefer not to have any (more) children?<br><br>IF SHE IS PREGNAT (CHECK A-10)<br>After the child you are expecting now, would you like to have (a/another) child, or would you prefer not to have any (more) children?	☐0 No more<br>☐1 Have (a/another) child<br>☐88 Undiceided/Don't know	→A-16<br><br>→A-16
A-15	Are you currently trying to get pregnant?	☐0 No<br>☐1 Yes	
A-16	Have you ever had an unwanted pregnancy?	☐0 No<br>☐1 Yes	
A-17	Have you ever had an abortion?	☐0 No<br>☐1 Yes	
A-18	How often have you had drinks containing alcohol during **the last 4 weeks**?	☐0 Less than once a week or never<br>☐1 At least once a week<br>☐2 Every day<br>☐88 Don't know	
A-19	Have you injected drugs in **the last 12 months**?<br>DRUGS INJECTED FOR MEDICAL PURPOSES DO NOT COUNT	☐0 No<br>☐1 Yes<br>☐88 Don't know<br>☐99 No response	

B) CONTRACEPTIVE USE

	Questions	Responses	Skip
B-1	Are you currently doing something or using any method **to delay or avoid getting pregnant** or **to prevent sexually transmitted infections and HIV**?	☐0 No<br>☐1 Yes	→B-20
B-2	Which method have you used for **the last 90 days**?<br>**MULTIPLE ANSWER IS POSSIBLE**<br>**CIRCLE ALL MENTIONED**	☐1 Female sterilization<br>☐2 Male sterilization<br>☐3 IUD<br>☐4 Injectables<br>☐5 Implants<br>☐6 Pill<br>☐7 Male condom<br>☐8 Female condom<br>☐9 Lactational amenorrhea method<br>☐10 Rhythm method<br>☐11 Withdrawal<br>☐12 Other method ( )	
B-3	Since what month and year have you been using (HIGHEST METHOD ON LIST IN B-2) without stopping?<br>*If the method is male or female condom, skip this question.	Month .... ☐☐<br>☐88 Don't know month<br>Year .... ☐☐☐☐<br>☐88 Don't know year	

B-4	Where did you get or learn (HIGHEST METHOD ON LIST IN B-2) at that time?	**Public sector**<br>☐1 Government hospital<br>☐2 Government health center<br>☐3 Government dispensary<br>**Private medical sector**<br>☐4 Private hospital/ clinic<br>☐5 Pharmacy/ Store<br>☐6 Nursing/Maternity home<br>☐7 Faith-based, Church, Mission hospital / Clinic<br>☐8 Family options/ fhok clinic<br>**Other sourse**<br>☐9 Shop<br>☐10 Community-based distributor<br>☐11 Community health worker<br>☐12 Friend/ Relative<br>☐13 **Other** ( )	
B-5	Why did you choose the method at that time?<br>Any others?<br>**RECORD WHAT SHE SAID**	.<br>.<br>.<br>☐88 Don't know<br>☐99 No response	
B-6	At that time, were you informed by a health care provider the extent to which the method provides protection against HIV and other STIs?	☐0 No<br>☐1 Yes<br>☐88 Don't know	
B-7	Does the method provide protection against HIV and other STIs?	☐0 No<br>☐1 Yes<br>☐88 Don't know	
B-8	**IF MORE THAN ONE METHOD SELECTED ON LIST IN B-2. IF NO, JUMP TO B-18**<br>Since what month and year have you been using (SECOND HIGHEST METHOD ON LIST IN B-2) without stopping?<br>*If the method is male or female condom, skip this question.	Month .... ☐☐<br>☐88 Don't know month<br>Year .... ☐☐☐☐<br>☐88 Don't know year	
B-9	Where did you get or learn (SECOND HIGHEST METHOD ON LIST IN B-2) at that time?	**Public sector**<br>☐1 Government hospital<br>☐2 Government health center<br>☐3 Government dispensary<br>**Private medical sector**<br>☐4 Private hospital/ clinic<br>☐5 Pharmacy/ Store<br>☐6 Nursing/Maternity home<br>☐7 Faith-based, Church, Mission hospital / Clinic<br>☐8 Family options/ fhok clinic<br>**Other sourse**<br>☐9 Shop<br>☐10 Community-based sistributor<br>☐11 Community health worker<br>☐12 Friend/ Relative<br>☐13 **Other** ( )	
B-10	Why did you choose the method at that time?<br>Any others?<br>**RECORD WHAT SHE SAID**	.<br>.<br>.<br>☐88 Don't know<br>☐99 No response	
B-11	At that time, were you informed by a health care provider the extent to which the method provides protection against HIV and other STIs?	☐0 No<br>☐1 Yes<br>☐88 Don't know	
B-12	Does the method provide protection against HIV and other STIs?	☐0 No<br>☐1 Yes	
B-13	**IF MORE THAN TWO METHODS SELECTED ON LIST IN B-2. IF NO, JUMP TO B-18**<br>Since what month and year have you been using (THIRD HIGHEST METHOD ON LIST IN B-2) without stopping?<br>*If the method is male or female condom, skip this question.	Month .... ☐☐<br>☐88 Don't know month<br>Year .... ☐☐☐☐<br>☐88 Don't know year	

B-14	Where did you get or learn (THIRD HIGHEST METHOD ON LIST IN B-2) at that time?	**Public sector**<br>□1 Government hospital<br>□2 Government health center<br>□2 Government dispensary<br>**Private medical sector**<br>□4 Private hospital/ clinic<br>□5 Pharmacy/ Store<br>□6 Nursing/Maternity home<br>□7 Faith-based, Church, Mission hospital / Clinic<br>□8 Family options/ fhok clinic<br>**Other sourse**<br>□9 Shop<br>□10 Community-based sistributor<br>□11 Community health worker<br>□12 Friend/ Relative<br>□13 **Other** ( )	
B-15	Why did you choose the method at that time?<br>Any others?<br>**RECORD WHAT SHE SAID**	·<br>·<br>·<br>□88 Don't know<br>□99 No response	
B-16	At that time, were you informed by a health care provider the extent to which the method provides protection against HIV and other STIs?	□0 No<br>□1 Yes<br>□88 Don't know	
B-17	Does the method provide protection against HIV and other STIs?	□0 No<br>□1 Yes	
B-18	Would you say that using contraception is mainly your decision, mainly your partner's decision, or did you both decide together?	□1 Mainly respondent<br>□2 Mainly husband/partner<br>□3 Joint decision<br>□4 Other ( )	
B-19	Does **your husband or live-in sexual partner** know you are using a method of family planning?	□0 No<br>□1 Yes<br>□88 Don't know<br>□89 No husband nor live-in sexual partner	→B-21<br>→B-21<br>→B-21<br>→B-21
B-20	What are reasons that you think you do not use a contraceptive method?<br>Any others?<br>**RECORD WHAT SHE SAID, THEN CIRCLE ALL ANSWERS THAT MATCH (IF ANY)**	·<br>·<br>·<br><br>□0 **Not married**<br>**Fertility-related reasons**<br>□1 Infrequent sex/no sex<br>□2 Menopausal/hysterectomy<br>□3 Subfecund/infecund<br>□4 Wants as many children as possible<br>**Opposition to use**<br>□5 Husband/partner opposed<br>□6 Others opposed<br>□7 Religious prohibition<br>**Lack of knowledge**<br>□8 Knows no method<br>□9 Knows no source<br>**Method-related reasons**<br>□10 Health concerns<br>□11 Fear of side effects<br>□12 Lack of access/too far<br>□13 Costs too much<br>□14 Inconvenient to use<br>□15 Interferes with body's normal processes<br>□88 Don't know<br>□99 No response	
B-21	Do you think that **your husband / live-in sexual partner** approves or disapproves of couples using a method to avoid pregnancy?	□0 Disapproves<br>□1 Approves<br>□88 Don't know<br>□89 No husband nor live-in sexual partner	

B-22	In **the last 12 months** have you:<br>a) Heard about family planning on the radio?<br>b) Seen anything about family planning on the television?<br>c) Read about family planning in a newspaper?<br>d) Heard about family planning by any staff at the health facility?<br>e) Heard about family planning by a community health worker?	NO YES<br>a) On the radio ☐0 ☐1<br>b) On the television ☐0 ☐1<br>c) On a newspaper ☐0 ☐1<br>d) By staff at the health facility ☐0 ☐1<br>e) By a community health worker ☐0 ☐1	
B-23	Have you ever *heard* of a male or female condom?	☐0 No<br>☐1 Yes	→C-1
B-24	Which places or persons do you know where you can obtain male or female condoms?<br>**MULTIPLE ANSWER IS POSSIBLE**<br>**CIRCLE ALL MENTIONED**	☐1 Shop<br>☐2 Pharmacy<br>☐3 Market<br>☐4 Hospital/Clinic/Health center/Dispensary<br>☐5 Bar/guest house/hotel<br>☐6 Community health worker<br>☐7 Friend<br>☐8 Other ( )<br>☐88 Don't know<br>☐99 No response	 <br><br><br><br><br><br><br>→C-1<br>→C-1
B-25	How long would it take you to obtain a male or female condom close to your house or to where you work?	☐1 Under 1 hour<br>☐2 1 hour to 3 hours<br>☐3 More than 3 hours<br>☐88 Don't know	

C) SEXUAL ACTIVITIES

	Questions	Responses	Skip
C-1	At what age did you first have sexual intercourse?	Age in completed years..................... ☐☐<br>☐88 Don't know<br>☐99 No response	
C-2	Have you excanged or sold sex for money, favors, or goods in **the last 12 months**?	☐0 No<br>☐1 Yes<br>☐99 No response	
C-3	Think about the male sexual partners you've had in **the last 90 days**. How many were:<br>A) **Regular partner** (your spouse or live-in sexual partner)<br>B) **Non-regular partner (s**exual partners that you are not married to and not living with)<br>IF NONE, RECORD '00'.	A) **Regular partner (s) ....** ☐☐<br>☐88 Don't know<br>☐99 No response<br>B) **Non-regular partner (s) ....** ☐☐<br>☐88 Don't know<br>☐99 No response	
C-4	**FILTER: CHECK C-3**<br>Did you have sex with your regular partner during **the past 90 days**?	☐0 No<br>☐1 Yes<br>☐88 Don't know<br>☐99 No response	→C-11<br><br>→C-11<br>→C-11
C-5	Think about your most recent regular sexual partner. The last time you had sex with this regular partner, did you and your partner use a male or female condom?	☐0 No<br>☐1 Yes<br>☐88 Don't know<br>☐99 No response	→C-7<br><br>→C-8<br>→C-8
C-6	Who suggested condom use that time?<br>**CIRCLE ONE**	☐1 Myself<br>☐2 My partner<br>☐3 Joint decision<br>☐88 Don't know<br>☐99 No response	→C-8<br>→C-8<br>→C-8<br>→C-8<br>→C-8
C-7	Why didn't you and your partner use a male or female condom that time?<br>Any others?<br>**RECORD WHAT SHE SAID , THEN CIRCLE ALL ANSWERS THAT MATCH (IF ANY)**	.<br>.<br>.<br>.<br>☐1Not available<br>☐2Too expensive<br>☐3Partner objected<br>☐4Don't like them<br>☐5Used other contraceptive<br>☐6Didn't think it was necessary<br>☐7Didn't think of it<br>☐88 Don't know<br>☐99 No response	

C-8	With what frequency did you and all of your regular partner(s) use a male or female condom during **the past 90 days**?	☐$_{0}$ Never<br>☐$_{1}$ Sometimes<br>☐$_{2}$ Almost every time<br>☐$_{3}$ Every time<br>☐$_{88}$ Don't know<br>☐$_{99}$ No response	
C-9	Have you had sex with a regular partner when the condom has slipped off or beoken in **the last 12 months**?	☐$_{0}$ No<br>☐$_{1}$ Yes<br>☐$_{88}$ Don't know<br>☐$_{99}$ No response	
C-10	With what frequency did you have sexual intercourse with your regular partner(s) under the influence of alcohol or drugs during **the past 90 days**?	☐$_{0}$ Never<br>☐$_{1}$ Sometimes<br>☐$_{2}$ Almost every time<br>☐$_{3}$ Every time<br>☐$_{88}$ Don't know<br>☐$_{99}$ No response	
C-11	FILTER: CHECK C-3<br><br>Did you have sex with your non-regular partner during **the past 90 days**?	☐$_{0}$ No<br>☐$_{1}$ Yes<br>☐$_{88}$ Don't know<br>☐$_{99}$ No response	→D-1<br><br>→D-1<br>→D-1
C-12	Think about your most recent non-regular sexual partner. The last time you had sex with this non-regular partner, did you and your partner use a male or female condom?	☐$_{0}$ No<br>☐$_{1}$ Yes<br>☐$_{88}$ Don't know<br>☐$_{99}$ No response	→C-14<br><br>→C-15<br>→C-15
C-13	Who suggested condom use that time?<br>**CIRCLE ONE**	☐$_{1}$ Myself<br>☐$_{2}$ My partner<br>☐$_{3}$ Joint decision<br>☐$_{88}$ Don't know<br>☐$_{99}$ No response	→C-15<br>→C-15<br>→C-15<br>→C-15<br>→C-15
C-14	Why didn't you and your partner use a male or female condom that time?<br>Any others?<br>**RECORD WHAT SHE SAID , THEN CIRCLE ALL ANSWERS THAT MATCH (IF ANY)**	·<br>·<br>·<br>·<br><br>☐$_{1}$Not available<br>☐$_{2}$Too expensive<br>☐$_{3}$Partner objected<br>☐$_{4}$Don't like them<br>☐$_{5}$Used other contraceptive<br>☐$_{6}$Didn't think it was necessary<br>☐$_{7}$Didn't think of it<br>☐$_{88}$ Don't know<br>☐$_{99}$ No response	
C-15	With what frequency did you and all of your non-regular partner(s) use a male or female condom during **the past 90 days**?	☐$_{0}$ Never<br>☐$_{1}$ Sometimes<br>☐$_{2}$ Almost every time<br>☐$_{3}$ Every time<br>☐$_{88}$ Don't know<br>☐$_{99}$ No response	
C-16	Have you had sex with a non-regular partner when the condom has slipped off or beoken in **the last 12 months**?	☐$_{0}$ No<br>☐$_{1}$ Yes<br>☐$_{88}$ Don't know<br>☐$_{99}$ No response	
C-17	With what frequency did you have sexual intercourse with your non-regular partner(s) under the influence of alcohol or drugs during **the past 90 days**?	☐$_{0}$ Never<br>☐$_{1}$ Sometimes<br>☐$_{2}$ Almost every time<br>☐$_{3}$ Every time<br>☐$_{88}$ Don't know<br>☐$_{99}$ No response	

D) STIs

	Questions	Responses	Skip
D-1	Have you ever heard of diseases that can be transmitted through sexual intercourse?	☐0 No<br>☐1 Yes	→D-3
D-2	Can you describe any symptoms of STDs in women? ..... Any others?<br><br>DO NOT READ OUT THE SYMPTOMS<br>CHECK NO FOR ALL NOT MENTIONED. CHECK YES FOR ALL MENTIONED.	NO / YES<br>a) Abdominal pain ☐0 ☐1<br>b) Genital discharge ☐0 ☐1<br>c) Foul smelling discharge ☐0 ☐1<br>d) Burning pain on urination ☐0 ☐1<br>e) Genital ulcers/ sores ☐0 ☐1<br>f) Swellings in groin area ☐0 ☐1<br>g) Itching ☐0 ☐1<br>h) Other ( ) ☐0 ☐1	
D-3	Have you had an abnormal genital discharge during **the past 12 months**?	☐0 No<br>☐1 Yes<br>☐88 Don't know<br>☐99 No response	
D-4	Have you had a genital ulcer/sores during **the past 12 months**?	☐0 No<br>☐1 Yes<br>☐88 Don't know<br>☐99 No response	

E) HIV

	Questions	Responses	Skip
E-1	Have you ever heard of HIV or the disease called AIDS?	☐0 No<br>☐1 Yes	→E-12
E-2	Can people protect themselves from HIV by using a condom correctly every time they have sex?	☐0 No<br>☐1 Yes<br>☐88 Don't know	
E-3	Can a person get HIV from mosquito bites?	☐0 No<br>☐1 Yes<br>☐88 Don't know	
E-4	Can people protect themselves from HIV by having one uninfected faithful sex partner?	☐0 No<br>☐1 Yes<br>☐88 Don't know	
E-5	Can people protect themselves from HIV by abstaining from sexual intercourse?	☐0 No<br>☐1 Yes<br>☐88 Don't know	
E-6	Can a person get HIV by sharing a meal with someone who is infected?	☐0 No<br>☐1 Yes<br>☐88 Don't know	
E-7	Can a person get HIV by getting injections with a needle that was already used by someone else?	☐0 No<br>☐1 Yes<br>☐88 Don't know	
E-8	Do you think that a healthy-looking person can be infected with HIV, the virus that causes AIDS?	☐0 No<br>☐1 Yes<br>☐88 Don't know	
E-9	Can a pregnant woman infected with HIV or AIDS transmit the virus to her unborn child?	☐0 No<br>☐1 Yes<br>☐88 Don't know	→E-11<br><br>→E-11

E-10	What can a pregnant woman do to reduce the risk of transmission of HIV to **her unborn child**?	NO YES<br>a) Take medication (Antiretrovirals) ☐0 ☐1<br>b) Visit ANC ☐0 ☐1<br>c) Other ( ) ☐0 ☐1<br>☐88 Don't know<br>☐99 No response	
E-11	Can a woman with HIV or AIDS transmit the virus to her newborn child through breastfeeding?	☐0 No<br>☐1 Yes<br>☐88 Don't know	
E-12	*Now we would like to ask you some personal questions about HIV status. Please understand that this interview is completely confidential and your answers will never be revealed to anyone. You do not need to answer the following question if you do not want.*<br><br>Have you ever been told by a health care provider that you have HIV?	☐0 No<br>☐1 Yes<br>☐88 Don't know<br>☐99 No response	→E-14<br><br>→E-14<br>→E-14
E-13	Are you receiving receiving antiretroviral therapy?	☐0 No<br>☐1 Yes<br>☐99 No response	
E-14	Does **your husband/live-in sexul partner** know your HIV status?	☐0 He doesn't know<br>☐1 He knows<br>☐88 Don't know<br>☐89 No husband nor live-in sexual partner<br>☐99 No response	 <br><br>→F-1
E-15	Do you know **your husband's or live-in sexual partner's** HIV status?	☐0 No<br>☐1 Yes<br>☐99 No response	→F-1<br><br>→F-1
E-16	What is his HIV status?	☐0 Negative<br>☐1 Positive<br>☐99 No response	→F-1<br><br>→F-1
E-17	Is he receiving receiving antiretroviral therapy?	☐0 No<br>☐1 Yes<br>☐88 Don't know<br>☐99 No response	

F) PERCEIVED RISK OF HIV SCALE

DO NOT ASK ANY QUESTIONS OF THIS SECTION FOR THOSE WHO REPLAYED THAT THEY HAVE BEEN TOLD THAT THAY HAVE HIV IN E-12

CHECK E-12: WHAT IS HER ANSWER? CIRCLE ONE
YES or NO RESPONSE (→G-1) / NO / DON'T KNOW

Now you will be asked your own feelings toward HIV risk.
Please check one response that best describes your own feelings using the scales below .

F-1	What is your gut feeling about how likely you are to get infected with HIV?	☐0 Extremely unlikely<br>☐1 Very unlikely<br>☐2 Somewhat likely<br>☐3 Very likely<br>☐4 Extremely likely	
F-2	I worry about getting infected with HIV	☐0 None of the time<br>☐1 Rarely<br>☐2 Some of the time<br>☐3 A moderate amount of time<br>☐4 A lot of the time<br>☐5 All of the time	
F-3	Picturing self getting HIV is something I find:	☐0 Very hard to do<br>☐1 Hard to do<br>☐2 Easy to do<br>☐3 Very easy to do	
F-4	I am sure I will NOT get infected with HIV	☐0 Strongly disagree<br>☐1 Disagree<br>☐2 Somewhat disagree<br>☐3 Somewhat agree<br>☐4 Agree<br>☐5 Strongly agree	
F-5	I feel vulnerable to HIV infection	☐0 Strongly disagree<br>☐1 Disagree<br>☐2 Somewhat disagree<br>☐3 Somewhat agree<br>☐4 Agree<br>☐5 Strongly agree	
F-6	There is a chance, no matter how small, I could get HIIV	☐0 Strongly disagree<br>☐1 Disagree<br>☐2 Somewhat disagree<br>☐3 Somewhat agree<br>☐4 Agree<br>☐5 Strongly agree	
F-7	I think my chances of getting infected with HIV are:	☐0 Zero<br>☐1 Almost zero<br>☐2 Small<br>☐3 Moderate<br>☐4 Large<br>☐5 Very Large	
F-8	Getting HIV is something I have	☐0 Never thought about<br>☐1 Rarely thought about<br>☐2 Thought about some of the time<br>☐3 Thought about often	

F-9	Do you think your chances of getting AIDS are small, moderate, great, no risk at all or don't you know?	☐0 No risk at all<br>☐1 Small<br>☐2 Moderate<br>☐3 Great<br>☐88 Don't know	

G) SELF-EFFICACY FOR CONDOM USE

This is the last part of the questionnaire.
Now you will be asked your own feelings toward male condom use. Please check one response that best describes your own feelings using scale ranging from "Strongly disagree" to "Strongly agree".

		Strongly disagree	Disagree	Undecided	Agree	Strongly agree
**G-1**	I feel confident in my ability to discuss condom usage with any partner I might have	□$_0$	□$_1$	□$_2$	□$_3$	□$_4$
**G-2**	I feel confident in my ability to suggest using condoms with a new partner	□$_0$	□$_1$	□$_2$	□$_3$	□$_4$
**G-3**	I feel confident I could suggest using a condom without my partner feeling "diseased"	□$_0$	□$_1$	□$_2$	□$_3$	□$_4$
**G-4**	I feel confident in my ability to persuade a partner to accept using a condom when we have sex	□$_0$	□$_1$	□$_2$	□$_3$	□$_4$
**G-5**	I would not feel confident suggesting using condoms with a new partner because I would be afraid he would think I've had a homosexual experience	□$_0$	□$_1$	□$_2$	□$_3$	□$_4$
**G-6**	I would not feel confident suggesting using condoms with a new partner because I would be afraid he would think I have a sexually transmitted disease	□$_0$	□$_1$	□$_2$	□$_3$	□$_4$
**G-7**	I would not feel confident suggesting using condoms with a new partner because I would be afraid he would think I thought they had a sexually transmitted disease	□$_0$	□$_1$	□$_2$	□$_3$	□$_4$
**G-8**	I feel confident that I would remember to use a condom even after I have been drinking	□$_0$	□$_1$	□$_2$	□$_3$	□$_4$
**G-9**	I feel confident that I would remember to use a condom even if I were high	□$_0$	□$_1$	□$_2$	□$_3$	□$_4$

**G-10**	Do you think you can convince your spouse or live-in sexual partner to use a condom during sex?	□$_0$ No<br>□$_1$ Yes<br>□$_{88}$ Don't know<br>□$_{89}$ No spouse nor live-in sexual partner

H) DUAL PROTECTION

PLEASE CHECK **B-2** AND **C-5**.

B-2: Has she used male/female condom with other methods in the last 90 days ? CIRCLE ONE
YES / NO

C-5: Did she use male/female condom with her regular partner at the last sex? CIRCLE ONE
YES / NO / DON'T KNOW / NO RESPONSE

IF THE ANSWERS TO B-2 AND C-5 ARE BOTH **YES**, ASK THE FOLLOWING QUESTION.

H-1 Many people do not use a condom with their spouse or live-in sexual partner, especially when thay are using another family planing method.
Why do you use condoms with your spouse or live-in sexual partner?
ANY OTHER REASONS?

Note:
・If she mentions about protection against both unwantend pregnancy and HIV/STIs, ask where, when and by whom she got this knowledge.
・If she mentioned about source of information only (like health provider told me), ask what kind of information she got from them.

The questionnaire is completed.
THANK YOU VERY MUCH FOR YOUR PARTICIPATION.
