# Supplementary material for: Consistent condom use among highly effective contraceptive users in an HIV-endemic area in rural Kenya
Source: PLoS One. 2019 May 6;14(5):e0216208. doi: 10.1371/journal.pone.0216208 (PMC6502455; doi:10.1371/journal.pone.0216208)
Supplement: S2 File — (PDF) [file pone.0216208.s008.pdf]

NO. □-□□□

DODOSO

Tarehe: D D / M M / Y Y Y Y
Hospitali:
Mhoji:
Mhariri:

A) TAARIFA ZA MSINGI

	Maswali	Majibu	Pita
A-1	Una miaka mingapi? (Miaka kamili)	Miaka kamili............................ □□<br>□88 Sijui	
A-2	Wewe ni kabila gani?	□1 Mjaluo<br>□2 Mkisii<br>□3 Mluhya<br>□4 Mkalenjin<br>□5 Mkikuyu<br>□6 Mengine ( )	
A-3	Je, dini yako ni gani?	□1 Mkatoliki<br>□2 Mrotestanti/Mkristo<br>□3 Muislamu<br>□4 Sina dini<br>□5 Mengine ( )	
A-4	Umefika kiwango kipi cha shule mwisho?	□0 Sijawai<br>□1 Sikumaliza shule ya msingi<br>□2 Nimemaliza shule ya msingi<br>□3 Sikumaliza shule ya upili<br>□4 Nimemaliza shule ya upili<br>□5 Zaidi ya shule ya upili	
A-5	Unafanya kazi gani?	□0 Sina kazi<br>□1 Ukulima<br>□2 Mfanyikazi wa serikali<br>□3 Sekta binafsi<br>□4 Biashara yangu binafsi<br>□5 Mwanafunzi<br>□6 Mengine ( )	
A-6	Je, nyumba yako ina:<br>a) Stima ?<br>b) Redio?<br>c) Runinga?<br>d) Simu ya mkono?<br>e) Simu ya meza/ya ofisi?<br>f) Frigi?<br>g) Jopo nishati ya jua?<br>h) Meza?<br>i) Kiti?<br>j) Sofa?<br>k) Kitanda?<br>l) Kabati?<br>m) Saa ya ukuta?<br>n) Mikrowevu?<br>o) Mchezaji DVD?<br>p) Mchezaji CD/Mkanda mchezaji?<br>q) Gari/Lovi?<br>r) Baiskeli?	NO YES<br>a) Stima □0 □1<br>b) Redio □0 □1<br>c) Runinga □0 □1<br>d) Simu ya mkono □0 □1<br>e) Simu ya meza/ya ofisi □0 □1<br>f) Frigi □0 □1<br>g) Jopo nishati ya jua □0 □1<br>h) Meza □0 □1<br>i) Kiti □0 □1<br>j) Sofa □0 □1<br>k) Kitanda □0 □1<br>l) Kabati □0 □1<br>m) Saa ya ukuta □0 □1<br>n) Mikrowevu □0 □1<br>o) Mchezaji DVD □0 □1<br>p) Mchezaji CD/Mkanda mchezaji □0 □1<br>q) Gari/Lovi □0 □1<br>r) Baiskeli □0 □1	
A-7	Nini hadi yako ya ndoa?	□1 Sijaolewa<br>□2 Nimeolewa<br>□3 Mjane<br>□4 Talaka	
A-8	Je, kwa sasa umeolewa ama unaishi na mume ambaye muna uhusiano wa kingono?	□1 Kwa sahii nimeolewa, naishi na mume<br>□2 Kwa sahii nimeolewa, naishi na mume mwingine ambaye tunashiriki ngono<br>□3 Kwa sahii nimeolewa, siishi na mume wangu wala mume yeyote tunayeshiriki ngono<br>□4 Sijaolewa, naishi na mume tunayeshiriki ngono<br>□5 Sijaolewa, siishi na mume wa kushiriki ngono<br>□99 Kimya	 <br><br>→A-10<br>→A-10

A-9	Je, mume/bwanako ana bibi/mbibi wengine?	☐0 La<br>☐1 Ndiyo<br>☐88 Sijui	
A-10	Uko mja mzito sai?	☐0 La<br>☐1 Ndiyo<br>☐2 Sina uhakika	
A-11	Umewai zaa?	☐0 La<br>☐1 Ndiyo	→A-14
A-12	Umewai zaa watoto wangapi?<br>KAMA HAKUNA, JAZA '00'.	.... ☐☐	
A-13	Ni watoto wako wangapi, wako hai?<br>KAMA HAKUNA, JAZA '00'.	.... ☐☐	
A-14	Ungependa kuwa na m/watoto wengine ama hunge penda kuwa na m/watoto yeyote?<br><br>KAMA MJA MZITO (ANGALIA A-10)<br>Baada ya huyu mtoto unayemtarajia sasa, ungependa kuwa na mw/wengine au la?	☐0 Sitaki wengine<br>☐1 Ningependa kuwa naye/nao<br>☐88 Sijaamua/Sijui	→A-16<br><br>→A-16
A-15	Unajaribu kupata mimba kwa sai?	☐0 La<br>☐1 Ndiyo	
A-16	Umewai pata mimba bila kutarajia au kupangia?	☐0 La<br>☐1 Ndiyo	
A-17	Umewai toa mimba?	☐0 La<br>☐1 Ndiyo	
A-18	Ni mara ngapi umekunyawa vinywaji zilizonapombe **kwa wiki minne** zilizopita?	☐0 Chini ya mara moja kwa wiki au sijawai<br>☐1 Mara moja kwa wiki<br>☐2 Kila siku<br>☐88 Sijui	
A-19	Umejidunga dawa **kwa miezi kumi na mbili** zilizopita?<br>MADAWA YALIYODUNGWA KWA AJILI YA MATIBABU USIHESABU	☐0 La<br>☐1 Ndiyo<br>☐88 Sijui<br>☐99 Kimya	

B) MATUMIZI YA NJIA ZA KUKINGA MIMBA

	Maswali	Majibu	Pita
B-1	Kwa saii, kuna chochote unachofanya au njia yoyote unayotumia kukinga kupata mimba au kukinga magonjwa ya zinaa na virusi vya ukimwi?	☐0 La<br><br>☐1 Ndiyo	→B-20
B-2	Ni njia lpi umetumia **kwa siku tisini** zilizopita?<br>**KUNA UWEZEKANO WA MAJIBU ZAIDI YA MOJA, TIA DUARA KWA ZOTE. ALIZOTAJA.**	☐1 Kufunya uzazi kwa mwanamke<br>☐2 Kufunya uzazi kwa mwanaume/Vasektomi<br>☐3 Kitanzi/Lupu<br>☐4 Sindano ya uzazi<br>☐5 Vipandikizi<br>☐6 Tembe/Vidonge<br>☐7 Mpira ya wanaume/ Kondom za kiume<br>☐8 Mpira ya wanawake/ Kondom za kike<br>☐9 Kunyonyesha<br>☐10 Kusoma kalenda<br>☐11 Kushusha kando<br>☐12 Njia mengine ( )	
B-3	Tangu mwezi na mwaka gani umekuwa ukitumia njia hii(YA KWANZA KWENYE CHAGUO LA B-2) bila kuacha?<br>*Kama ni njia ya mpira ya mwanaume au kike, ruka swali hili.	Mwezi .... ☐☐<br>☐88 Sijui mwezi<br>Mwaka .... ☐☐☐☐<br>☐88 Sijui mwaka	

B-4	Ulipata au kujifunza wapi (NJIA YA KWANZA KATIKA ORODHA YA B-2) wakati huo?	**Matibu katika serikali**<br>☐1 Hospitali ya serikali<br>☐2 Kituo cha afya cha serikali<br>☐3 Zahanati ya serikali<br>**Matibu ya kibinafsi**<br>☐4 Hospitali/ Kliniki ya kibinafsi<br>☐5 Dula la dawa<br>☐6 Chumba cha uuguzi/uzaaji<br>☐7 Hospitali/ Kliniki ya kidini<br>☐8 Familia kuchangua/ Uchaguzi wa familia<br>**Mahala pengine**<br>☐9 Dukani<br>☐10 Jamii msingi distributor<br>☐11 Mhudumu wa afya katika jamii (CHW)<br>☐12 Rafiki/Jamaa<br>☐13 Mengine ( )	
B-5	Kwa nini ulichagua njia hiyo wakati huo?<br>Sababu nyingine?<br>**TANAKALI ALICHOSEMA**	.<br>.<br>.<br>☐88 Sijui<br>☐99 Kimya	
B-6	Wakati huo, ulikuwa umeelezewa na mhudumu wa afya kiwango ambacho njia hio inakinga kutokana na virusi vya ukimwi na magonjwa mengine ya zinaa?	☐0 La<br>☐1 Ndiyo<br>☐88 Sijui	
B-7	Je, njia hio inakinga kutokana na virusi vya ukimwi na magonjwa mengine ya zinaa?	☐0 La<br>☐1 Ndiyo<br>☐88 Sijui	
B-8	**IWAPO NJIA ZAIDI YA MOJA ZILICHAGULIWA KUTOKA B-2. KAMA LA, RUKA HADI B-18.**<br>Tongu mwezi na mwaka gani umekuwa ukitumia njia hii(YA PILI KWENYE CHAGUO LA B-2) bila kuacha?<br>*Kama ninjia ya mpira ya mwanaume au kike, ruka swali hili.	Mwezi .... ☐☐<br>☐88 Sijui mwezi<br>Mwaka .... ☐☐☐☐<br>☐88 Sijui mwaka	
B-9	Ulipata au kujifunza wapi (YA PILI KWENYE CHAGUO LA B-2) wakati huo?	**Matibu katika serikali**<br>☐1 Hospitali ya serikali<br>☐2 Kituo cha afya cha serikali<br>☐3 Zahanati ya seikali<br>**Matibu ya kibinafsi**<br>☐4 Hospitali/ Kliniki ya kibinafsi<br>☐5 Dula la dawa<br>☐6 Chumba cha uuguzi/uzaaji<br>☐7 Hospitali/ Kliniki ya kidini<br>☐8 Familia kuchangua/ Uchaguzi wa familia<br>**Mahala pengine**<br>☐9 Dukani<br>☐10 Jamii msingi distributor<br>☐11 Mhudumu wa afya katika jamii (CHW)<br>☐12 Rafiki/Jamaa<br>☐13 Mengine ( )	
B-10	Kwa nini ulichagua njia hiyo wakati huo?<br>Sababu nyingine?<br>**TANAKALI ALICHOSEMA**	.<br>.<br>.<br>☐88 Sijui<br>☐99 Kimya	
B-11	Wakati huo, ulikuwa umeelezewa na mhudumu wa afya kiwango ambacho njia hio inakinga kutokana na virusi vya ukimwi na magonjwa mengine ya zinaa?	☐0 La<br>☐1 Ndiyo<br>☐88 Sijui	
B-12	Je, njia hio inakinga kutokana na virusi vya ukimwi na magonjwa mengine ya zinaa?	☐0 La<br>☐1 Ndiyo<br>☐88 Sijui	
B-13	**IWAPO NJIA ZAIDI YA MBILI ZILICHAGULIWA KUTOKA B-2. KAMA LA, RUKA HADI B-18.**<br>Tongu mwezi na mwaka gani umekuwa ukitumia njia hii(YA TATU KWENYE CHAGUO LA B-2) bila kuacha?<br>*Kama ninjia ya mpira ya mwanaume au kike, ruka swali hili.	Mwezi .... ☐☐<br>☐88 Sijui mwezi<br>Mwaka .... ☐☐☐☐<br>☐88 Sijui mwaka	

B-14	Ulipata au kujifunza wapi (YA TATU KWENYE CHAGUO LA B-2) wakati huo?	**Matibu katika serikali**<br>☐1 Hospitali ya serikali<br>☐2 Kituo cha afya cha serikali<br>☐3 Zahanati ya seikali<br>**Matibu ya kibinafsi**<br>☐4 Hospitali/ Kliniki ya kibinafsi<br>☐5 Dula la dawa<br>☐6 Chumba cha uuguzi/uzaaji<br>☐7 Hospitali/ Kliniki ya kidini<br>☐8 Familia kuchangua/ Uchaguzi wa familia<br>**Mahala pengine**<br>☐9 Dukani<br>☐10 Jamii msingi distributor<br>☐11 Mhudumu wa afya katika jamii (CHW)<br>☐12 Rafiki/Jamaa<br>☐13 Mengine ( )	
B-15	Kwa nini ulichagua njia hiyo wakati huo?<br>Sababu nyingine?<br>**TANAKALI ALICHOSEMA**	·<br>·<br>·<br>☐88 Sijui<br>☐99 Kimya	
B-16	Wakati huo, ulikuwa umeelezewa na mhudumu wa afya kiwango ambacho njia hio inakinga kutokana na virusi vya ukimwi na magonjwa mengine ya zinaa?	☐0 La<br>☐1 Ndiyo<br>☐88 Sijui	
B-17	Je, njia hio inakinga kutokana na virusi vya ukimwi na magonjwa mengine ya zinaa?	☐0 La<br>☐1 Ndiyo<br>☐88 Sijui	
B-18	Waweza sema kuwa kutumia njia ya kukinga kupata mimba ni uamuzi wako, mume wako au nyinyi wote kwa pamoja?	☐1 Wangu mwenyewe<br>☐2 Wa bwanangu/ mume wangu<br>☐3 Uamuzi wetu wawili<br>☐4 Mengine ( )	
B-19	Je, **<u>mume/bwana yako</u>** anafahamu kuwa unatumia njia ya kupanga uzazi?	☐0 La<br>☐1 Ndiyo<br>☐88 Sijui<br>☐89 Sina mume/bwana	→B-21<br>→B-21<br>→B-21<br>→B-21
B-20	Ni sababu zipi unafikiria zinakufanya usitumie njia ya kuzuia kupata mimba?<br>Sababu nyingine?<br>**TANAKALI ALICHOSEMA**<br>**KISHA CHORA MAJIBU YOTE YANAYOAMBATANA (KAMA KUNAYO)**	·<br>·<br>·<br>☐0 **Not married**<br>**Fertility-related reasons**<br>☐1 Infrequent sex/no sex<br>☐2 Menopausal/hysterectomy<br>☐3 Subfecund/infecund<br>☐4 Wants as many children as possible<br>**Opposition to use**<br>☐5 Husband/partner opposed<br>☐6 Others opposed<br>☐7 Religious prohibition<br>**Lack of knowledge**<br>☐8 Knows no method<br>☐9 Knows no source<br>**Method-related reasons**<br>☐10 Health concerns<br>☐11 Fear of side effects<br>☐12 Lack of access/too far<br>☐13 Costs too much<br>☐14 Inconvenient to use<br>☐15 Interferes with body's normal processes<br>☐88 Don't know<br>☐99 No response	
B-21	Je, wafikiria **<u>mume/bwana yako</u>** anakubaliana nayo ama kupinga matumizi ya njia za kukinga mimba kwa wapendanao?	☐0 Anapinga<br>☐1 Anakubaliana<br>☐88 Sijui<br>☐89 Sina mume/bwana	

B-22	**Kwa miezi kumi na mbili** zilizopita umawai:<br>a) Kusikia kuhusu kupanga uzazi kwa redio?<br>b) Kuona chochote kuhusu kupanga uzazi kwa runinga?<br>c) Kusoma kuhusu kupanga uzazi kwa gazeti?<br>d) Kusikia kuhusu kupanga uzazi kutoka kwa mhudumu yeyote wa afya hospitalini?<br>e) Kusikia kuhusu kupanga uzazi kutoka kwa mkunga/daktari mdogo?	NO / YES<br>a) Kwa redio □0 □1<br>b) Kwa runinga □0 □1<br>c) Kwa gazeti □0 □1<br>d) Kutoka kwa mhudumu wa afya hospitalini □0 □1<br>e) Kutoka kwa muhudumu wa afya katika jamii (CHW) □0 □1	
B-23	Umewai sikia kuna mpira ya wanaume au kike?	□0 La<br>□1 Ndiyo	→C-1
B-24	Ni wapi au kwa nani unajua unaeza zipata mpira za kiume au kike?<br>**KUNA UWEZEKANO WA MAJIBU ZAIDI YA MOJA, TIA DUARA KWA ZOTE. ALIZOTAJA.**	□1 Dukani<br>□2 Duka la dawa<br>□3 Sokoni<br>□4 Hospitali/Kliniki<br>□5 Bar/guest house/hotelini<br>□6 Muhudumu wa afya katika jamii (CHW)<br>□7 Rafiki<br>□8 Mengine ( )<br>□88 Sijui<br>□99 Kimya	 <br><br><br><br><br><br><br>→C-1<br>→C-1
B-25	Unaweza kuchukua muda gani kuchukua mpira wa kike au kiume karibu na pale unakoishi ama unakofanya kazi?	□1 Chini ya saa moja<br>□2 Saa moja hadi masaa matatu<br>□3 Zaidi ya masaa matatu<br>□88 Sijui	

C) USHIRIKI WA NGONO

	Maswali	Majibu	Pita
C-1	Ulianza kushiriki ngono mwanzo ukiwa na miaka ngapi?	Miaka kamili...................... □□<br>□88 Sijui<br>□99 Kimya	
C-2	Umewai shiriki ngono kwa ajili ya kupewa pesa, kufanyiwa kizuri au kupewa bidhaa **kwa miezi kumi na miwili** iliopita?	□0 La<br>□1 Ndiyo<br>□99 Kimya	
C-3	Tafakari kuhusu wanaume wako wakushiriki ngono ambao umekuwa nawo **kwa siku tisin**i zilizopita.<br>Ni wangapi walikuwa:<br>A) **Wa kilamara** (mume/bwanako)<br>B) **Wasio wa kilamara** (wanaume mnaoshiriki ngono lakini hamjaona wala hamuishi naye)<br>KAMA HAKUNA, TANAKALI '00'.	A) **Mume wa kilamara** .... □□<br>□88 Sijui<br>□99 Kimya<br>B) **Wasio wa kilamara** .... □□<br>□88 Sijui<br>□99 Kimya	
C-4	**CHUJIO: ANGALIA C-3**<br>Umeshiriki ngono na mume wako wa kila mara au bwanako **kwa siku tisini** zilizopita?	□0 La<br>□1 Ndiyo<br>□88 Sijui<br>□99 Kimya	→C-11<br><br>→C-11<br>→C-11
C-5	Tafakari kuhusu yule mume wa mwisho kushiriki ngono naye. Mara ya mwisho mkishiriki ngono naye, je mlitumia mpira ya kiume au kike?	□0 La<br>□1 Ndiyo<br>□88 Sijui<br>□99 Kimya	→C-7<br><br>→C-8<br>→C-8
C-6	Ni nani alipendekeza kuhusu kutumia mpira wakati huo?	□1 Mimi mwenyewe<br>□2 Mume wangu<br>□3 Uamuzi wetu wawili<br>□88 Sijui<br>□99 Kimya	→C-8<br>→C-8<br>→C-8<br>→C-8<br>→C-8
C-7	Ni kwa nini wewe au mume wako hamkutumia mpira wa kike au kiume wakati huo?<br>Sababu nyinginezo?<br>**TANAKALI ALICHOSEMA**<br>**KISHA CHORA MAJIBU YOTE YANAYOAMBATANA (KAMA KUNAYO)**	.<br>.<br>.<br>.<br>□1Not available<br>□2Too expensive<br>□3Partner objected<br>□4Don't like them<br>□5Used other contraceptive<br>□6Didn't think it was necessary<br>□7Didn't think of it<br>□88 Don't know<br>□99 No response	

C-8	Ni mara ngapi **kwa siku tisini** zilizopita, wewe na mume/waume wako wa kila mara mlitumia mpira wa kike au kiume?	□$_{0}$ Hakuna<br>□$_{1}$ Wakati mwingine<br>□$_{2}$ Karibu kila wakati<br>□$_{3}$ Kila wakati<br>□$_{88}$ Sijui<br>□$_{99}$ Kimya	
C-9	Umewai shiriki ngono na mume wako ambapo mpira ulitoka au kukatika **kwa miezi kumi na mbili** zilizopita?	□$_{0}$ La<br>□$_{1}$ Ndiyo<br>□$_{88}$ Sijui<br>□$_{99}$ Kimya	
C-10	Ni mara ngapi mumeshiriki ngono na mume/waume wako akiwa amelewa **kwa siku tisini** zilizopita?	□$_{0}$ Hakuna<br>□$_{1}$ Wakati mwingine<br>□$_{2}$ Karibu kila wakati<br>□$_{3}$ Kila wakati<br>□$_{88}$ Sijui<br>□$_{99}$ Kimya	
C-11	**CHUJIO: ANGALIA C-3**<br><br>Umeshiriki ngono na mpenzi **kwa siku tisin**i zilizopita?	□$_{0}$ La<br>□$_{1}$ Ndiyo<br>□$_{88}$ Sijui<br>□$_{99}$ Kimya	→D-1<br><br>→D-1<br>→D-1
C-12	Tafakari kuhusu yule mpenzi mara ya mwisho kushiriki ngono naye, mlitumia mpira ya kiume au kike?	□$_{0}$ La<br>□$_{1}$ Ndiyo<br>□$_{88}$ Sijui<br>□$_{99}$ Kimya	→C-14<br><br>→C-15<br>→C-15
C-13	Ni nani alipendekeza kutumia mpira wakati huo?	□$_{1}$ Mimi mwenyewe<br>□$_{2}$ Mpenzi<br>□$_{3}$ Uamuzi wetu wawili<br>□$_{88}$ Sijui<br>□$_{99}$ Kimya	→C-15<br>→C-15<br>→C-15<br>→C-15<br>→C-15
C-14	Ni kwa nini wewe au mpenzi huyo hamkutumia mpira wa kike au kiume wakati huo?<br>Sababu nyinginezo?<br>**TANAKALI ALICHOSEMA KISHA CHORA MAJIBU YOTE YANAYOAMBATANA (KAMA KUNAYO)**	.<br>.<br>.<br>.<br><br>□$_{1}$Not available<br>□$_{2}$Too expensive<br>□$_{3}$Partner objected<br>□$_{4}$Don't like them<br>□$_{5}$Used other contraceptive<br>□$_{6}$Didn't think it was necessary<br>□$_{7}$Didn't think of it<br>□$_{88}$ Don't know<br>□$_{99}$ No response	
C-15	Ni mara ngapi **kwa siku tisini** zilizopita, wewe na mpenzi/wapenzi wako mlitumia mpira wa kike au kiume?	□$_{0}$ Hakuna<br>□$_{1}$ Wakati mwingine<br>□$_{2}$ Karibu kila wakati<br>□$_{3}$ Kila wakati<br>□$_{88}$ Sijui<br>□$_{99}$ Kimya	
C-16	Umewai shiriki ngono na mpenzi wako ambapo mpira ulitoka au kukatika **kwa miezi kumi na mbili** zilizopita?	□$_{0}$ La<br>□$_{1}$ Ndiyo<br>□$_{88}$ Sijui<br>□$_{99}$ Kimya	
C-17	Ni mara ngapi mumeshiriki ngono na mpenzi/wapenzi wako akiwa amelewa **kwa siku tisini** zilizopita?	□$_{0}$ Hakuna<br>□$_{1}$ Wakati mwingine<br>□$_{2}$ Karibu kila wakati<br>□$_{3}$ Kila wakati<br>□$_{88}$ Sijui<br>□$_{99}$ Kimya	

D) MAGONJWA YA ZINAA

	Maswali	Majibu	Pita
D-1	Umewai sikia magonjwa yanayo sababishwa kutokana na kushiriki ngono?	☐0 La<br>☐1 Ndiyo	→D-3
D-2	Waweza eleza dalili yoyote ya magonjwa haya kwa mwanamke?<br><br>USISOME DALILI<br>ANGALIA KILA ASIYOTAJA. CHORA DUARA KWA ZOTE ALIZOTAJA.	NO / YES<br>a) Maumivu chini ya tumbo ☐0 ☐1<br>b) Usaa sehemu ya siri ☐0 ☐1<br>c) Usaa wa harufu mbaya ☐0 ☐1<br>d) Uchungu wakati wa haja ndogo ☐0 ☐1<br>e) Vidonda sehemu ya siri ☐0 ☐1<br>f) Uvimbe katika sehemu ya siri ☐0 ☐1<br>g) Kujikuna ☐0 ☐1<br>h) Mengine ( ) ☐0 ☐1	
D-3	Umewai kuwa na uchafu usio wa kawaida katika sehemu ya siri **kwa miezi kumi na mbili** zilizopita?	☐0 La<br>☐1 Ndiyo<br>☐88 Sijui<br>☐99 Kimya	
D-4	Umewai kuwa na vidonda sehemu ya siri **kwa miezi kumi na mbili** zilizopita?	☐0 La<br>☐1 Ndiyo<br>☐88 Sijui<br>☐99 Kimya	

E) VIRUSI VYA UKIMWI

	Maswali	Majibu	Pita
E-1	Umawai sikia virusi vya ukimwi au ugonjwa wa ukimwi?	☐0 La<br>☐1 Ndiyo	→E-12
E-2	Watu wanaweza jikinga kutokana na virusi vya ukimwi kwa kutumia mpira visawa kila wakati wanapo shiriki ngono?	☐0 La<br>☐1 Ndiyo<br>☐88 Sijui	
E-3	Mtu anaweza pata virusi vya ukimwi akiumwa na mbu?	☐0 La<br>☐1 Ndiyo<br>☐88 Sijui	
E-4	Je, watu wanaweza jikinga kutokana na virusi vya ukimwi kwa kuwa na mume au mpenzi mmoja asiye na virusi na mwaminifu?	☐0 La<br>☐1 Ndiyo<br>☐88 Sijui	
E-5	Je, watu wanaweza jikinga kutokana na virusi wakiwa wamekataa kushiriki ngono?	☐0 La<br>☐1 Ndiyo<br>☐88 Sijui	
E-6	Je, mtu anaeza pata virusi vya ukimwi akikula pamoja na mtu aliye na virusi au ugonjwa huo?	☐0 La<br>☐1 Ndiyo<br>☐88 Sijui	
E-7	Je, mtu anaeza pata virusi hivi kwa kutumia sindano moja iliyotumika na mtu mwingine?	☐0 La<br>☐1 Ndiyo<br>☐88 Sijui	
E-8	Unafikiria kuwa mtu aliye na afya bora pia aweza ambukizwa virusi vya ukimwi?	☐0 La<br>☐1 Ndiyo<br>☐88 Sijui	
E-9	Je, kuna uwezekano wa mama mjamzito kumuambukiza mtoto wake **aliye tumboni** virusi vya ukimwi akiwa akonayo?	☐0 La<br>☐1 Ndiyo<br>☐88 Sijui	→E-11 (La)<br>→E-11 (Sijui)

E-10	Mama mjamzito anaweza fanya nini ili apunguze uwezekano wa kumwambukiza mwanawe **<u>aliye tumboni</u>**?	NO / YES<br>a) Ameze dawa ya virusi ☐0 ☐1<br>b) Atembelee kliniki ☐0 ☐1<br>c) Mengine ( ) ☐0 ☐1<br>☐88 Sijui<br>☐99 Kimya	
E-11	Je, mama aliye na virusi vya ukimwi anaweza pia kumwambukiza mwanawe akiwa anamnyonyesha?	☐0 La<br>☐1 Ndiyo<br>☐88 Sijui	
E-12	*Sasa tungependa kuuliza maswali kuhusu hali yako ya virusi. Tafadhali elewa kwamba majibu yako yatawekwa kwa siri na hakuna atakayejua. Hakuna haja ujibu maswali haya kama hautaki.*<br><br>Umewai ambiwa na mhudumu yeyote wa afya kuwa una virusi vya ukimwi?	☐0 La<br>☐1 Ndiyo<br>☐88 Sijui<br>☐99 Kimya	→E-14<br><br>→E-14<br>→E-14
E-13	Je, unapokea tiba ya kurefusha maisha?	☐0 La<br>☐1 Ndiyo<br>☐99 Kimya	
E-14	**<u>Bwana/Mume wako</u>** anajua hali yako ya virusi?	☐0 Hajui<br>☐1 Anajua<br>☐88 Sijui<br>☐89 Sina mume/bwana<br>☐99 Kimya	 <br><br>→F-1
E-15	Je, wajua hali ya mume au bwanako ya virusi?	☐0 La<br>☐1 Ndiyo<br>☐99 Kimya	→F-1<br><br>→F-1
E-16	Ni nini hali yake ya virusi vya ukimwi?	☐0 Hana virusi<br>☐1 Ana virusi<br>☐99 Kimya	→F-1<br><br>→F-1
E-17	Je, anapokea tiba ya kurefusha maisha?	☐0 La<br>☐1 Ndiyo<br>☐88 Sijui<br>☐99 Kimya	

F) UNAVYOJUA HATARI YA VIRISI VYA UKIMWI WADOGO

USIULIZE SWALI HILI KWA WALE WALIOSEMA AWALI KUWA WANA VIRUSI VYA UKIMWI KWA E-12

ANGALIA E-12: JIBU LAKE NI LIPI?
NDIYO / LA (→G-1) / LA / SIJUI

Sasa, utaulizwa kuhusu hisia zako kibinafsi kulingana na hatari za virusi vya ukimwi.
Tafadhali chagua jibu moja linaloeleza vema hisia zako kwa kutumia majibu yafuatayo.

F-1	Hisia yako ni gani kuhusu uwezekano wako kuambukizwa virusi vya ukimwi	☐0 Haiwezekani kabisa<br>☐1 Haiwezekani<br>☐2 Saa zingine inawezekana<br>☐3 Inawezekana<br>☐4 Inawezekana sana	
F-2	Nina hofu kupata virusi vya ukimwi	☐0 Hakuna wakati wowote<br>☐1 Kwa nadra<br>☐2 Wakati mwingine<br>☐3 Kwa kiwango sawa tu<br>☐4 Mara nyingi<br>☐5 Kila wakati	
F-3	Kujiweka katika picha ya kupata virusi naipata kuwa:	☐0 Vigumu sana<br>☐1 Vigumu<br>☐2 Rahisi<br>☐3 Rahisi sana	
F-4	Nina uhakika kuwa sitaambukizwa ukimwi	☐0 Kataa kata<br>☐1 Kataa<br>☐2 Sina uhakika<br>☐3 Nina uhakika kidogo<br>☐4 Nakubali<br>☐5 Nakubali kabisa	
F-5	Mimi kujiskia mazingira magumu na virusi vya ukimwi	☐0 Kataa kata<br>☐1 Kataa<br>☐2 Sina uhakika<br>☐3 Nina uhakika kidogo<br>☐4 Nakubali<br>☐5 Nakubali kabisa	
F-6	Kuna uwezekano hata kama kidogo kivipi, naezapata virusi vya ukimwi	☐0 Kataa kata<br>☐1 Kataa<br>☐2 Sina uhakika<br>☐3 Nina uhakika kidogo<br>☐4 Nakubali<br>☐5 Nakubali kabisa	
F-7	Ninafikiri nafasi yangu ya kuambukizwa virusi vya ukimwi ni:	☐0 Sufuri<br>☐1 Karibu na sufuri<br>☐2 Kidogo<br>☐3 Kiasi<br>☐4 Kubwa<br>☐5 Kubwa sana	
F-8	Kuambukizwa ukimwi ni kitu	☐0 Sijawahi fikiria<br>☐1 Ni nadra kufikiria<br>☐2 Nimewahi fikiria mara nyingine<br>☐3 Nimefikiria mara nyingi	

F-9	Je, unafikiri vyanzo vyako vya kuambukizwa virusi ni kidogo, kiasi, kubwa, hakuna hatari au hujui?	☐0 Hakuna hatari kabisa<br>☐1 Kidogo<br>☐2 Kiasi<br>☐3 Kubwa<br>☐88 Sijui	

G) UFANISI BINAFSI KWA AJILI YA MATUNZI YA MPIRA

Huu ndio sehemu ya mwisho wa dodoso hili
Sasa, utaulizwa hisia zako binafsi kuhusu matumizi ya mpira za wanaume.Tafadhali chagua jibu moja linaloeleza kikamilifu hisia zako kwa kutumia mizani ya"sikubaliani sana" hadi "nakubali sana" .

		Sikubaliani sana	Sikubaliani	Sina uhakika	Nakubali	Nakubali sana
**G-1**	Nina ujasiri katika uwezo wangu kutoa maoni kuhusu matumizi ya mpira na mpenzi wangu yeyote	□$_0$	□$_1$	□$_2$	□$_3$	□$_4$
**G-2**	Nina ujasiri kwa uwezo wangu kutoa maoni kuhusu matumizi ya mpira na mpenzi mpya	□$_0$	□$_1$	□$_2$	□$_3$	□$_4$
**G-3**	Nina ujasiri kupendekeza kutumia mpira bila hisia za mpenzi wangu kuguswa kuwa "mgonjwa"	□$_0$	□$_1$	□$_2$	□$_3$	□$_4$
**G-4**	Nina ujasiri katika uwezo wangu wa kumshawishi mpenzi kukubali kutumia mpira wakati tunashiriki ngono	□$_0$	□$_1$	□$_2$	□$_3$	□$_4$
**G-5**	Sijiskii mjasiri kupendekeza kutumia mpira na mpenzi mpya kwa sababu atafikiria niliwaihusiana na uzoefu mashoga	□$_0$	□$_1$	□$_2$	□$_3$	□$_4$
**G-6**	Sijiskii mjasiri kupendekeza kutumia mpira na mpenzi mpya kwa sababu nina hofu atadhani nina magonjwa ya zinaa	□$_0$	□$_1$	□$_2$	□$_3$	□$_4$
**G-7**	Sijiskii mjasiri kupendekeza kutumia mpira na mpenzi mpya kwa sababu nina uwoga kuwa atafikiria ninadhani ana magonjwa ya zinaa	□$_0$	□$_1$	□$_2$	□$_3$	□$_4$
**G-8**	Nina ujasiri kuwa nitaweza kumbuka kutumia mpira hata baada ya kunywa pombe	□$_0$	□$_1$	□$_2$	□$_3$	□$_4$
**G-9**	Nina ujasiri kuwa nitaweza kumbuka kutumia mpira hata kama nilikuwa na hamu ya mwanaume	□$_0$	□$_1$	□$_2$	□$_3$	□$_4$

**G-10**	Je, unafikiria una uwezo wa kumshawishi mumeo au mapenzi mnayeshiriki ngono naye kutumia mpira wakati wa ngono	□$_0$ La<br>□$_1$ Ndiyo<br>□$_{88}$ Sijui<br>□$_{89}$ Sina mume/bwana

H) KINGA MBILI

TAFADHALI ANGALIA B-2 NA **C-5**.

B-2: Amewaitumia mpira pamoja na njia zinginezo kwa siku 90 zilizopita?
NDIYO / LA

C-5: Je, alitumia mpira pamoja na yule mume/bwana wakati walishiriki ngono mara ya mwisho?
NDIYO / LA / HAJUI / KIMYA

IWAPO MAJIBU YA B-2 NA C-5 NI NDIYO, ULIZA SWALI LIFUATALO.

H-1 Watu wengi hawatumii mpira wakishiriki ngono na waume wao, sanasana wakiwa wanatumia njia nyingine ya kupanga uzazi. Je, mbona wewe watumia mpira pamoja na mume wako/bwana?
SABABU NYINGINEZO?

Kumbuka:
・Ikiwa ametaja kuhusu kinga ya mimba na ukimwi na ugonjwa ya zinaa, uliza ni wapi, lini na kwa nani alipata ujumbe huo.
・Ikiwa ametaja kuhusu alipopata ujumbe(kwa mfano kuambiwa na mhudumu wa afya), uliza ni ujumbe upi, haswa alipata kutoka kwa mhudumu huyo.

DODOSO LIMEISHA.
AHSANTE SANA KWA USHIRIKA WAKO.
